# Differentially private federated learning for localized control of infectious disease dynamics

**DOI:** 10.1038/s41598-026-60725-1

**Published:** 2026-07-06

**Authors:** Raouf Kerkouche, Henrik Zunker, Mario Fritz, Martin J. Kühn

**Affiliations:** 1 CISPA Helmholtz Center for Information Security, Saarbrücken, Germany; 2 Department of High-Performance Computing, Institute of Software Technology, German Aerospace Center, Cologne, Germany; 3 Bonn Center for Mathematical Life Sciences and Life and Medical Sciences Institute, University of Bonn, Bonn, Germany

**Keywords:** Federated learning, Differential privacy, Infectious disease forecasting, COVID-19, Machine learning, Public health, MEmilio, Diseases, Health care, Mathematics and computing

## Abstract

In times of epidemics, swift reaction is necessary to mitigate epidemic spreading. For this reaction, localized approaches have several advantages, limiting necessary resources and reducing the impact of interventions on a larger scale. However, training a separate machine learning (ML) model on a local scale is often not feasible due to limited available data. Centralizing the data is also challenging because of its high sensitivity and privacy constraints. In this study, we consider a localized strategy based on the German counties and communities managed by the related local health authorities (LHA). For the preservation of privacy to not oppose the availability of detailed situational data, we propose a privacy-preserving forecasting method that can assist public health experts and decision makers. ML methods with federated learning (FL) train a shared model without centralizing raw data. Considering the counties, communities or LHAs as clients and finding a balance between utility and privacy, we study a FL framework with client-level differential privacy (DP). We train a shared multilayer perceptron on sliding windows of recent case counts to forecast the number of cases in the future, while clients exchange only norm-clipped updates and the server aggregates updates with DP noise. We evaluate the approach on COVID-19 data on county-level during two phases: November 2020 and March 2022 (Omicron). As expected, very strict privacy ($\varepsilon \le 0.5$) yields unstable, unusable forecasts. At a moderately strong but still privacy-preserving level ($\varepsilon = 2$), the DP model closely approaches the non-DP model: $R^2\approx 0.93$ (vs. 0.96) and mean absolute percentage error (MAPE) $\approx 26\%$ in November 2020; $R^2\approx 0.85$ (vs. 0.90) and MAPE $\approx 24\%$ in March 2022. Overall, our results support the feasibility of privacy-preserving collaboration among health authorities for local forecasting. In the evaluated COVID-19 phases, client-level DP-FL delivered useful county-level predictions with formal privacy guarantees under the stated threat model. The appropriate privacy budget should nevertheless be re-evaluated for other epidemic phases and applications.

## Introduction

In epidemic emergencies or situations of seasonally peaking endemic pathogens, public health experts and decision makers can benefit from predictions of infectious disease dynamics to proactively react to upcoming challenges. For prediction tasks, mechanistic mathematical models as well as ML models have proven to provide reliable outcomes. While for mechanistic population^1–3^, metapopulation^4,5^, agent-based^6–8^ or combined differential equation- and ML-based^9,10^ approaches, new situations might require the adaptations of existing mechanisms, many ML models are developed in a purely data-driven fashion, reducing the number of manual interventions cycles. In particular, classical simulation or forecasting studies^1–3,7,8^ mostly used publicly available datasets and did not consider the aspect of privacy-preservation.

The traditional approach to training machine learning models typically requires centralizing data from multiple parties. This creates serious privacy risks when handling sensitive information such as medical records, which often cannot be shared directly. In infectious disease contexts, these records include precise dates of symptom onset (essential for estimating reporting delays), assumed transmission events, and detailed demographics maintained by hospitals or LHAs. These features are critical for predicting future cases but are removed from public datasets to preserve anonymity. While aggregation reduces disclosure risk, it removes these valuable signals and does not eliminate the risk of re-identification^11^. Ensuring privacy-preserving approaches is therefore essential when working with such data.

To train ML models on spatially distributed data, FL has been introduced, enabling the collaborative training of a shared model without the need for data centralization^12–14^. FL allows each client to train the model on their own data and share only the model updates, or gradients, through a central server. The server aggregates these updates to improve the shared model, which is then redistributed to participants for further refinement. This process repeats until the model achieves satisfactory performance.

FL is grounded in three key objectives: First, it aims to protect the privacy of each participant’s data by exchanging model updates rather than the data itself. Second, it seeks to lower communication costs by allowing several local training iterations before the exchange of updates. Finally, by involving only a subset of clients in each round, it reduces communication overhead and increases resilience to the temporary unavailability of clients, offering particular advantages in scenarios with a large number of clients.

However, sharing gradients during collaborative learning can still reveal sensitive information from the training data of individual parties. Advanced attacks have demonstrated that adversaries can infer specific data records or group characteristics from intercepted model updates^15–22^. In extreme cases, they can reconstruct entire training samples from the gradients^20,21^. Therefore, merely avoiding the sharing of actual training data cannot completely avoid privacy leakage.

Initial works on calibrating noise to sensitivity^23^ led to an extensive work of algorithmic foundations of *Differential Privacy*^24^. DP has emerged as a preeminent framework for enhancing data privacy, countering various privacy attacks associated with FL. DP provides strong privacy assurances by ensuring that the behavior of the aggregated model does not depend significantly on any single client’s data. Instead, it captures commonalities across all contributions. In the context of federated learning, several foundational works have combined $(\varepsilon ,\delta )$-DP with standard federated learning algorithms that aggregate client updates on a central server^25–27^, or with local differential privacy mechanisms in which each client perturbs its model update before sending it to the server^28^. An overview of the federated learning landscape, including privacy preservation, is given by^29^. Implementing DP in these settings typically involves clipping client updates to a fixed sensitivity and adding Gaussian noise during aggregation, which helps protect participant-specific information while preserving overall learning utility. In 2021, Ficek et al.^30^ observed a substantial increase in the use of DP in health research publications, and frameworks for privacy-preserving FL in the health domain have been developed in parallel^31–34^. In the context of the COVID-19 pandemic, Hauer and Santos-Lozada^35^ raised awareness for a potential distortion of COVID-19 death rates when using DP with too small dataset groupings. During the COVID-19 pandemic, more than 1.9 million COVID-19 cases across Europe have been integrated in the DataSHIELD (Data Aggregation Through Anonymous Summary-statistics from Harmonised Individual-levEL Databases) platform^36^, to allow for FL approaches to derive potential strategies against COVID-19^37^. Among other things, DataSHIELD has already been used for other infectious diseases, e.g., to study malaria intervention in Mozambique^38^.

In order to report dynamics and allow predictions by simultaneously addressing privacy concerns, COVID-19 case counts had been aggregated and reported on county-level throughout the pandemic in Germany^39^. We use these official aggregates as a proof-of-concept dataset to evaluate our method, which is intended for settings where finer-grained, potentially sensitive data remain with the LHAs. While aggregation reduces disclosure risk, it does not provide formal privacy guarantees^11^. Our work builds on established algorithms for federated learning with differential privacy^25,26,28^ and selected DP-FL mechanism. To allow the reliable application in a public health context, we focus on a realistic infectious disease forecasting use case to study and evaluate client-level DP-FL on county-level COVID-19 data from two distinct epidemic phases in Germany. Selecting an established setup in which clients send clipped model updates and the server adds Gaussian noise calibrated to a target $(\varepsilon ,\delta )$-DP guarantee via a Rényi DP accountant, we map out the privacy-utility tradeoff across a broad grid of privacy budgets and identify ranges of $\varepsilon$ for which forecasts remain practically useful. The novelty of this work lies not in the existence of the privacy-utility tradeoff, which is well-established theoretically, but in its systematic, quantitative characterization for county-level epidemic forecasting: (i) we evaluate two epidemiologically distinct phases to assess robustness across different dynamics; (ii) we map the tradeoff across a fine-grained $\varepsilon$ grid and quantify the transition from unusable to practically useful predictions; and (iii) we provide actionable guidance for public health practitioners on viable privacy budgets for short-term forecasting.

## Results

To evaluate the feasibility of localized, privacy-preserving forecasting, we applied a DP-FL framework to predict short-term COVID-19 case numbers. Specifically, we train a shared multilayer perceptron (MLP) on historical case counts to predict future infections. In this setup, clients exchange only norm-clipped updates, which a central server aggregates with Gaussian noise to provide formal privacy guarantees under the stated threat model. Detailed formalisms of our training pipeline, model architecture, and privacy accounting are provided in the Methods section.

We first present the predictive performance of our approach applied to real-world county-level data during two distinct epidemic phases and analyze the resulting privacy-utility tradeoff. Subsequently, we discuss preliminary findings for finer-resolved synthetic community data.

### Dataset

**Fig. 1:**
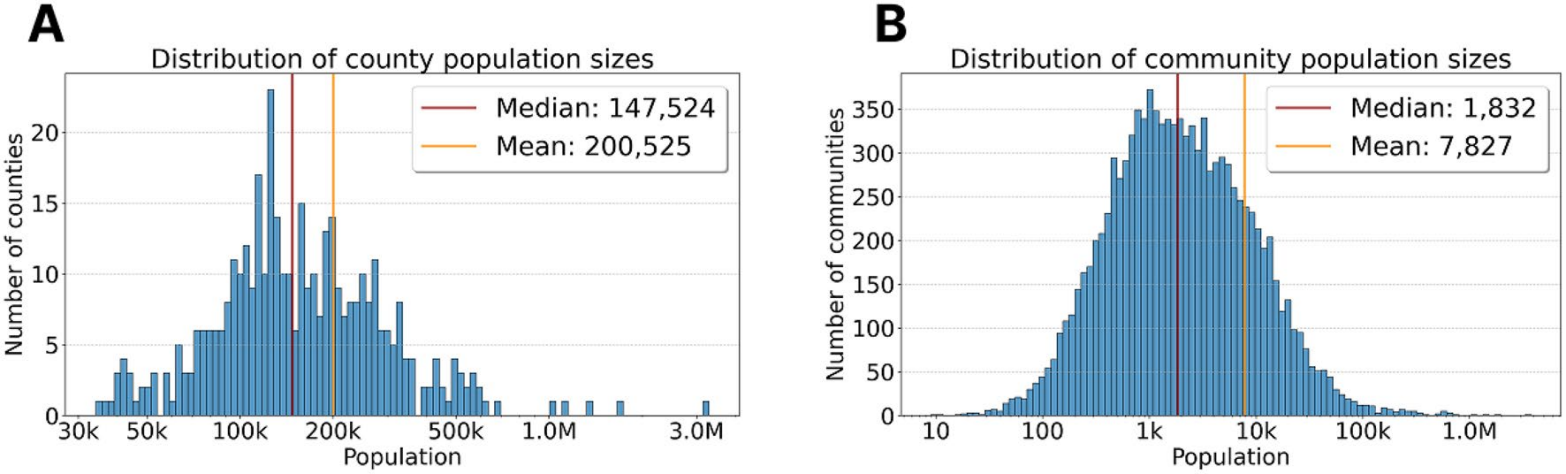
Distribution of population sizes on community and county-level. (**A**) Shows the distribution of county population sizes across all 400 German counties, with a median population of 147,524 and mean population of 200,525. (**B**) Displays the distribution of community population sizes across the 10,786 communities included in our analysis, with a substantially smaller median population of 1,832 and mean of 7,827. Red and orange vertical lines indicate median and mean values, respectively.

We use official reported county-level COVID-19 case data from the LHAs transferred to the Robert Koch Institute (RKI)^39^ and accessed via the *memilio-epidata* Python package^40^. We consider two different pandemic stages, first, late-stage as of March 2022 and, second, early stage as of November 2020. Throughout this work, the November 2020 data and March 2022 data refer to two separate one-month windows of the county-level COVID-19 cases data. Models are trained and evaluated independently on each period. For each county and day, we obtain the single attribute used in this study: the newly confirmed infections reported with the (assumed) day of infection. No additional attributes such as demographics, hospitalizations, or test positivity rates are used. This data covers all 400 German counties across two one-month study windows: November 2020 (early-stage exponential growth) and March 2022 (late-stage Omicron wave). While the published data is stratified on county-level, the counties can, again, be divided by communities, summing up to a total of 10,786 communities in Germany. In Fig. 1A shows the population distribution across the counties, highlighting the variation in population sizes. The median county population is 147,524, while the mean population is 200,525, as several counties have a substantially larger population. The largest county by population has 3,685,265 inhabitants (Berlin, after merging all Berlin districts), and the total population across all counties is approximately 83.6 million. This heterogeneity in county sizes presents a particular challenge for our FL approach. Counties with larger populations typically report higher absolute case numbers. In addition, we show in Panel B the distribution of population sizes on community-level.

#### Synthetic community data

To examine whether predictive performance is maintained on finer levels and to evaluate robustness, we generated synthetic case datasets on community-level in Germany. In Fig. 1, we show the distribution of population sizes for community and county sizes. While the median county population is 147,524 as of 2022, the median community population is 1,832; see^41^.

**Fig. 2:**
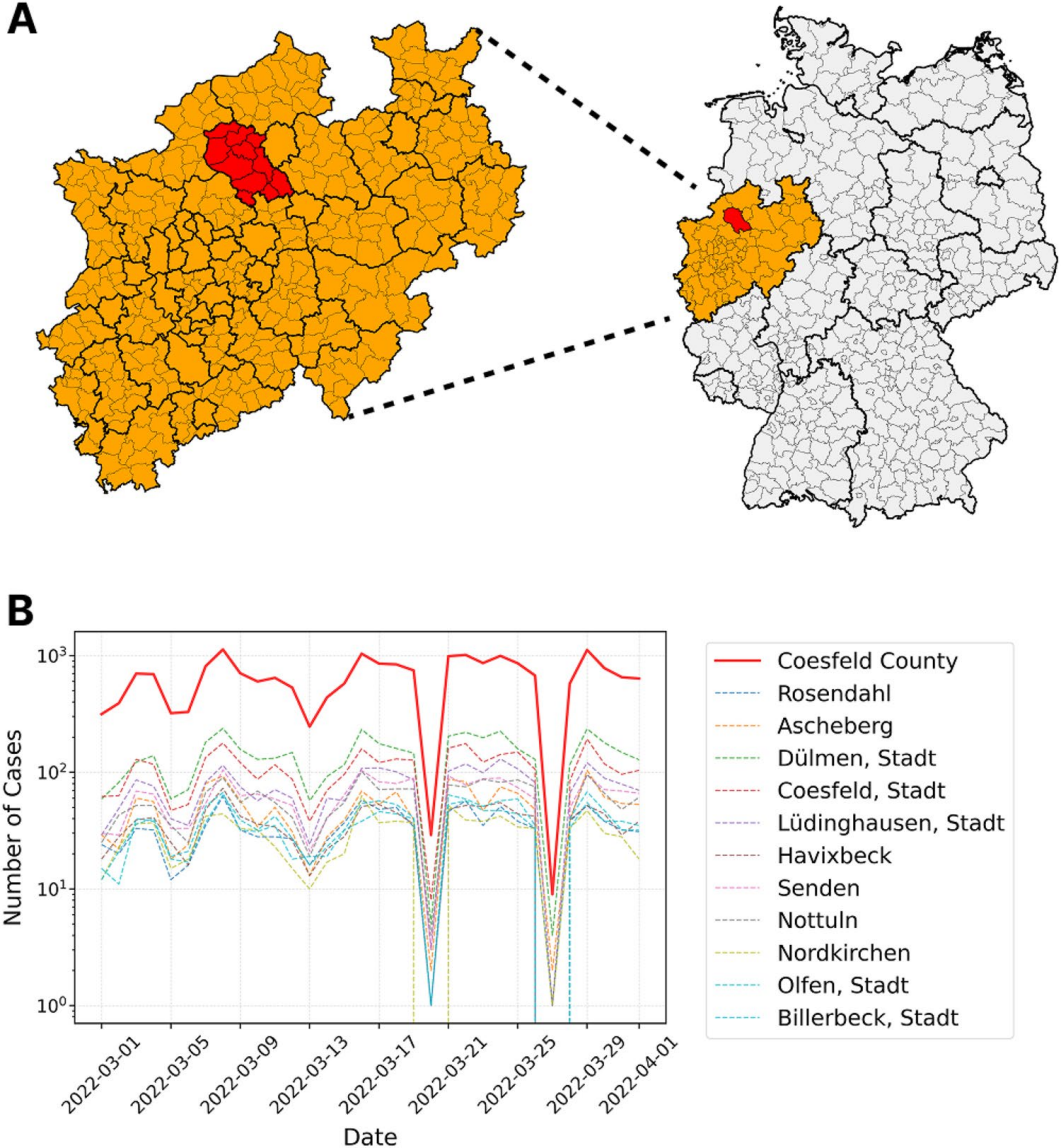
Spatial resolution of German counties and communities and community-based case data. (**A**) Presents two maps shown side by side. The left map presents North Rhine-Westphalia (NRW) stratified into counties (thick black boundaries) and communities (thin gray boundaries), highlighting county ”Coesfeld” in red. The right map displays the stratification of Germany into federal states (thick black boundaries) and counties (thin gray boundaries), highlighting the federal state NRW in orange. (**B**) Shows the obtained community-based dataset for ”Coesfeld” for March 2022, with the county-level aggregate shown in red and individual community trajectories in various colors. The left map from Panel A is using geodata “Verwaltungsgebiete 1:250 000 (VG250)” from BKG (2026) dl-de/by-2-0, Data sources: https://sgx.geodatenzentrum.de/web_public/gdz/datenquellen/datenquellen_vg_nuts.pdf.

To generate a community-based dataset, we sampled randomly from the county-based dataset, using weights given by community population divided by the corresponding county population. For the spatial stratification and an exemplary result for the county of ”Coesfeld”, see Fig. 2B. In the resulting dataset, zeros were imputed for days when no case data was sampled for the particular community.

Based on this synthetic dataset, we applied our DP-FL approach. Instead of reporting the results directly, which, when measured in MAPE or $R^2$, looked much better than they actually were, we provide preliminary analyses on the outcomes. In particular, when DP was used (noise added to the aggregated, clipped updates), we did not observe deterioration of predictions, which we interpret as a cautionary signal.

**Fig. 3:**
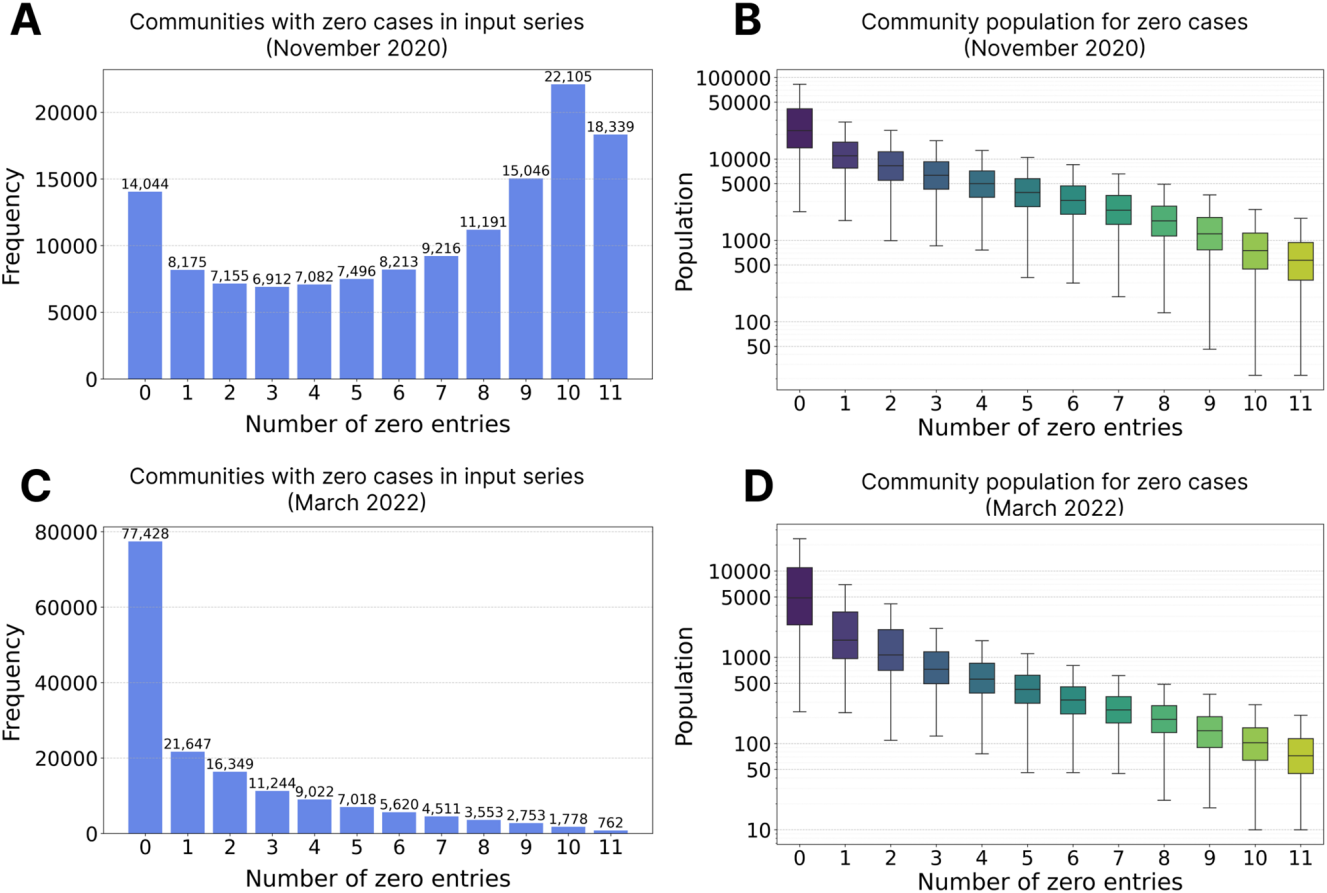
Communities with zero cases on input horizon and prediction day. (**A**) Shows the number of communities with 0 to 11 zero entries in the considered time series for November 2020 and (**B**) shows the size of the community populations with the corresponding number of zeros. (**C**,**D**) Report the same structure for March 2022.

In Fig. 3, we provide the number of communities with zero confirmed cases over the input horizon $H=10$ days plus the one prediction day; cf. Eqs. (1) and (2). While we see that for March 2022 far fewer communities have a high number of zero entries, the approach showed similar problems for both periods. With the application of a 7-day trailing moving average, we substantially reduced the number of zero entries but did not obtain a dataset for which we observed substantially different results. That means, we did not observe a prediction deterioration when the noise increased with $\varepsilon \rightarrow 0.3$. We suspect that this outcome is an artifact due to two properties. In the synthetically created dataset, we obtain either relatively many zero entries for a large number of community time series or very small entries in the case of a moving average applied. In addition, the random sampling for the community dataset could have yielded time series which were already highly noisy such that the addition of DP noise did not qualitatively change the time series pattern to be learned. In order for FL and DP to be validated on community-level, we conclude that additional experiments will be necessary and that access to a true community-based dataset will be required.

#### Published county data

**Fig. 4:**
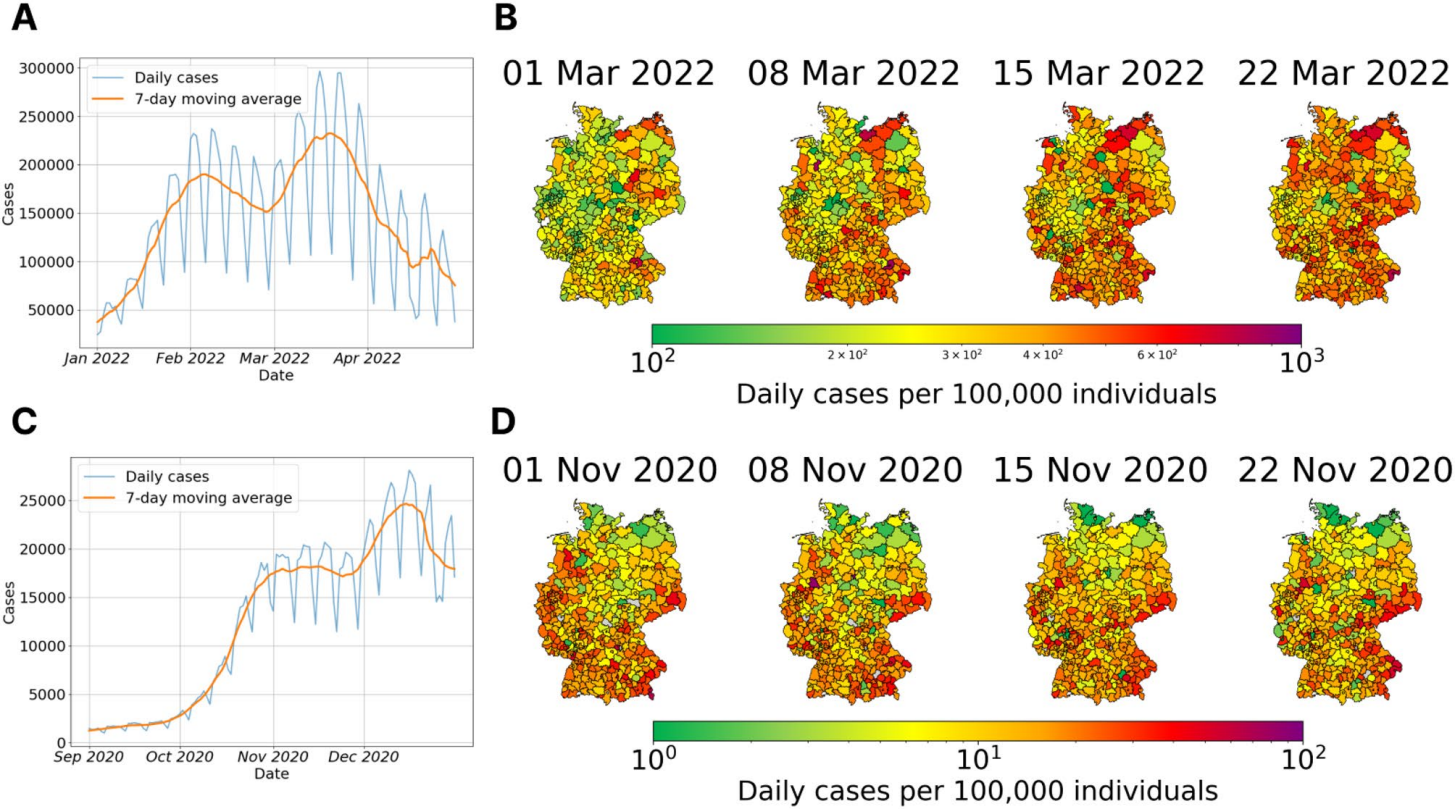
Analysis of COVID-19 case numbers in Germany on county-level. (**A**) Presents the time series of daily new infections throughout early 2022, showing both raw daily counts (blue) and the trailing 7-day moving average (orange) that smooths the weekly reporting pattern. (**B**) plots the geographical distribution of the COVID-19 incidence per 100,000 population at four weekly intervals in March 2022, illustrating the spatial heterogeneity of infection patterns and their temporal evolution. (**C**,**D**) show the same type of data for late 2020.

As county-level case data was the finest resolved data available, we validate our approach on this level. In Fig. 4A,C, we see the total infection dynamics in Germany with clearly visible waves of infection during early 2022 and late 2020. These Panels also demonstrate the typical weekly reporting pattern observed in the data, with pronounced drops in reported cases during weekends followed by increases during weekdays. To mitigate these reporting artifacts, we applied a trailing 7-day moving average to the daily case counts, resulting in smoother time series as shown by the orange line.

In Fig. 4B,C, we present the geographical distribution of COVID-19 cases across the German counties. We see that the local entities have their own infection dynamics and that waves are partly desynchronized.

### County-level prediction March 2022

**Fig. 5:**
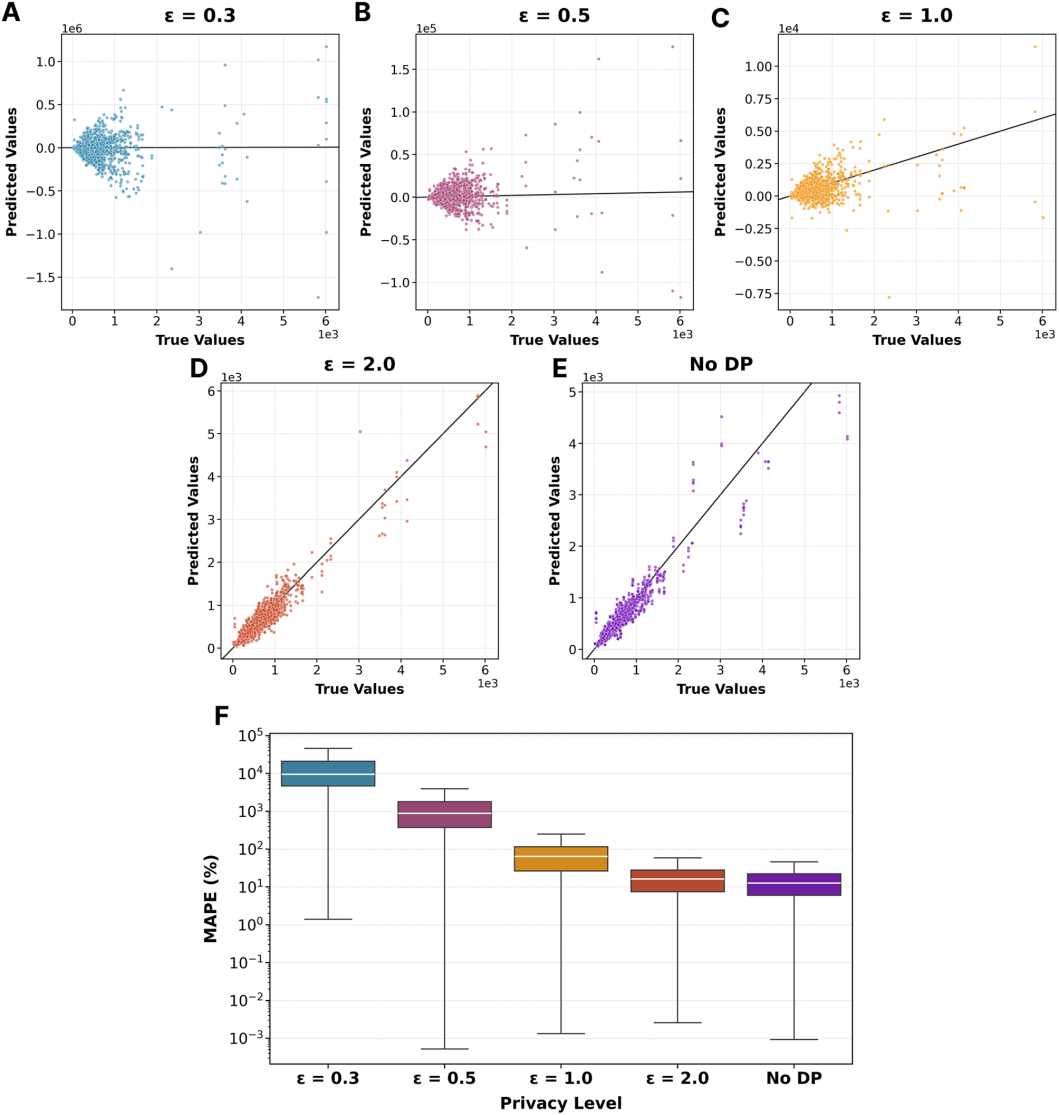
Comparison of prediction performance across different privacy levels for March 2022 data. (**A**–**E**) Show scatter plots of predicted against true case counts for various privacy budgets: (**A**) $\varepsilon = 0.3$, (**B**) $\varepsilon = 0.5$, (**C**) $\varepsilon = 1.0$, (**D**) $\varepsilon = 2.0$, and (**E**) $\varepsilon = \infty$ (non-DP). The black diagonal line represents perfect prediction. (**F**) Displays box plots of MAPE for individual county predictions across all runs and privacy levels, illustrating the decreasing error and variance as privacy budget increases.

First, we evaluated our FL approach on county-level COVID-19 case data from March 2022, which represents a challenging period during the Omicron wave characterized by high peaks and dynamic infection trajectories. In Table 1 and Fig. 5, we present the performance metrics across different privacy budget levels, ranging from strict privacy guarantees ($\varepsilon = 0.3$) to no differential privacy ($\varepsilon = \infty$, non-DP).

**Table 1:** Performance metrics for COVID-19 case prediction on March 2022 data. Results from 15 runs, 75 federated rounds, and 30 local epochs per round for different privacy budget levels. The metrics include Mean Squared Error (MSE), Mean Absolute Error (MAE), (MAPE), and $R^2$ score. All values are reported as mean ± standard deviation across runs.

$\varepsilon$	MSE	MAE	MAPE (%)	$R^2$
0.3	$9.97 \times 10^{9} \pm 2.41 \times 10^{10}$	$4.97 \times 10^{4} \pm 5.47 \times 10^{4}$	$9.62 \times 10^{3} \pm 1.04 \times 10^{4}$	$-3.75 \times 10^{4} \pm 9.07 \times 10^{4}$
0.5	$1.12 \times 10^{8} \pm 1.24 \times 10^{8}$	$6.37 \times 10^{3} \pm 4.66 \times 10^{3}$	$1.23 \times 10^{3} \pm 8.75 \times 10^{2}$	$-4.20 \times 10^{2} \pm 4.67 \times 10^{2}$
1.0	$7.21 \times 10^{5} \pm 1.19 \times 10^{6}$	$460.49 \pm 416.71$	$89.94 \pm 76.83$	$-1.72 \pm 4.50$
2.0	$3.88 \times 10^{4} \pm 2.19 \times 10^{4}$	$114.74 \pm 35.30$	$23.62 \pm 6.69$	$0.85 \pm 0.08$
non-DP	$2.52 \times 10^{4} \pm 1.30 \times 10^{3}$	$87.69 \pm 2.22$	$18.15 \pm 0.36$	$0.90 \pm 0.00$

Our results demonstrate a clear privacy-utility tradeoff that is characteristic of DP implementations. As shown in Fig. 5, the quality of predictions declines substantially as we strengthen the privacy guarantees, i.e., by decreasing the privacy budget $\varepsilon$. In particular, for $\varepsilon = 0.3$ and $\varepsilon = 0.5$ the models are effectively unusable as the predictions show no correlation with the true case counts (see Panel A and B in Fig. 5). At the strictest privacy level ($\varepsilon = 0.3$), the model produced predictions that were almost completely unrelated to the actual values, with an extremely high MSE of $9.97 \times 10^{9}$ and a strongly negative $R^2$ value of approximately $-37{,}500$. The MAPE was over 9,600%, which makes the complete failure of the predictive ability even more clear. Also, the standard deviation of these metrics across the runs is of the same order of magnitude or larger than the mean values themselves, indicating extreme instability in the prediction behavior. At $\varepsilon = 0.5$, while still poor, the model begins to show marginal improvement, with MSE reduced by about two orders of magnitude to $1.12 \times 10^8$. However, the predictions remain basically unusable with MAPE near 1,200% and $R^2$ still deeply negative at approximately $-420$. Fig. 5B shows that while there is slightly more structure to the predictions, they still fail to track the true values in any meaningful way. A substantial qualitative improvement emerges at $\varepsilon = 1.0$, where the MSE drops to around 721,000, and MAPE decreases to approximately 90%. While the $R^2$ value remains negative at $-1.72$, indicating the model still performs worse than a simple mean predictor, the predictions begin to show some correlation with the true values, as shown in Fig. 5C. At $\varepsilon = 2.0$, we observe that the model achieves positive predictive value, with an $R^2$ of 0.85. Despite the privacy, the overall performance is comparable to the non-DP case. The MAPE decreases to around 24%, and the predictions visibly track the true values as shown in Fig. 5D. The non-private (non-DP) model shows our performance ceiling with an $R^2$ value of 0.90 and the lowest MAPE at 18.15%. Fig. 5E demonstrates the most accurate tracking of true values.

Beyond the mean performance metrics, the standard deviations across runs reveal important insights about the stability of differentially private models. As $\varepsilon$ decreases, the standard deviations increase dramatically, reflecting the noise introduced by the privacy mechanism. This is especially evident in Fig. 5F, which shows box plots of the MAPE calculated for individual county predictions. For these box plots, the individual MAPE values for each county from all runs were collected and then visualized separately for each privacy level. Each box plot thus represents the distribution of prediction errors across all counties and runs for a given privacy level.

The substantial outliers for $\varepsilon = 0.3$ and $\varepsilon = 0.5$ illustrate the extreme variability in model performance at these privacy levels, whereas the non-DP and $\varepsilon = 2.0$ cases show much more consistent performance.

**Fig. 6:**
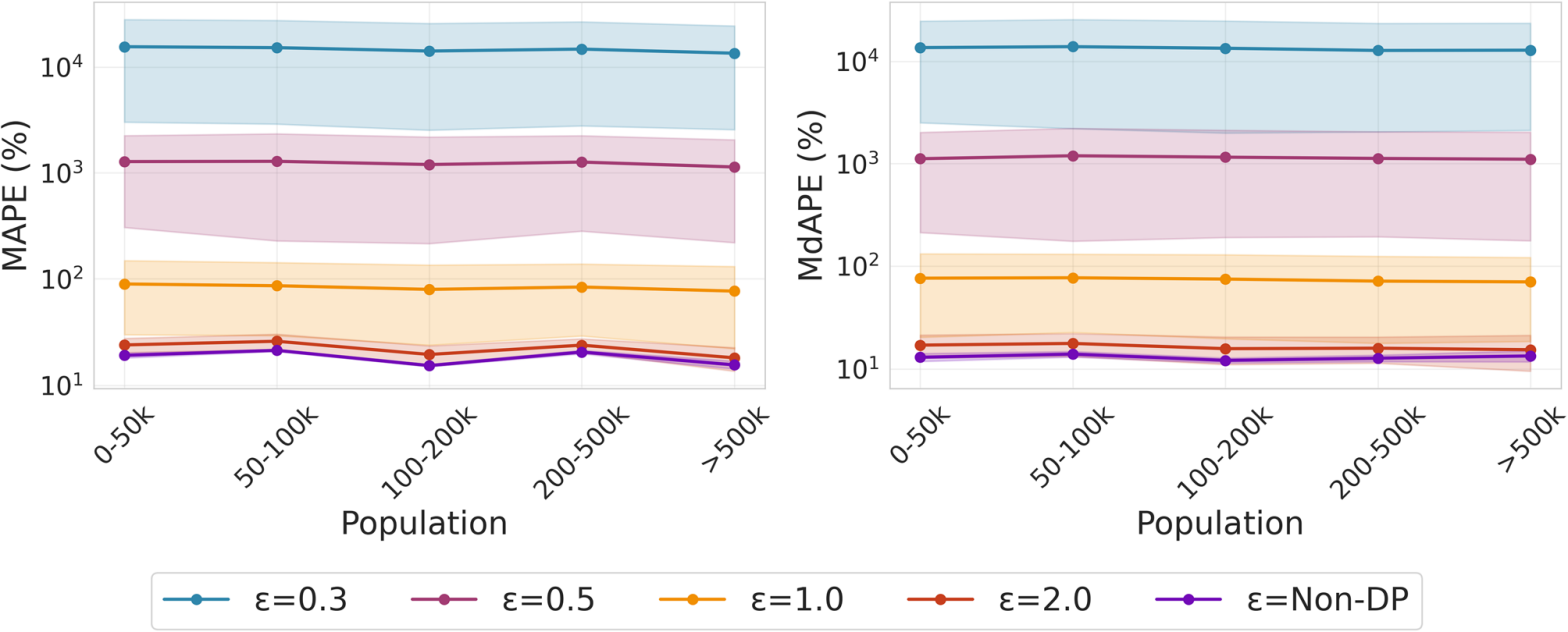
Prediction performance stratified by county population size. The panels show MAPE and Median Absolute Percentage Error (MdAPE) for different privacy levels ($\varepsilon \in \{0.3, 0.5, 1.0, 2.0\}$ and non-DP) across five population clusters (0–50k, 50–100k, 100–200k, 200–500k, >500k inhabitants), calculated from 15 runs, 75 federated rounds and 30 local epochs per round. The shaded areas represent the standard deviation across all 15 runs.

To additionally analyze the impact of population heterogeneity, we stratified the test samples into five population clusters (0–50k, 50–100k, 100–200k, 200–500k, >500k). Figure 6 shows the relative performance metrics (MAPE and MdAPE) for each cluster. While absolute errors naturally scale with population size, these relative metrics mainly remain stable across all clusters. This suggests that smaller counties are not substantially disadvantaged by the global model. Instead, they likely benefit from the shared learning of temporal patterns driven by data-rich counties, which stabilizes predictions on their potentially sparser local data.

These results highlight that for our dataset, there appears to be a minimum viable privacy budget (approximately $\varepsilon = 1.0$) below which predictions become too unreliable for practical use. However, the model at $\varepsilon = 2.0$ demonstrates that it is possible to nearly match the performance of a non-DP model while retaining a formal client-level DP guarantee under the stated threat model. This suggests that when designing privacy-preserving prediction systems for infectious disease dynamics, the balance of privacy requirements against the need for reliable predictions must be carefully considered.

### County-level prediction November 2020

In addition, we evaluate our model’s performance and the DP tradeoff for an earlier stage of the pandemic as of November 2020. The November 2020 data evaluation reveals patterns consistent with those observed in the 2022 dataset, but with several notable differences that highlight how pandemic phase and disease dynamics influence the effectiveness of privacy-preserving predictive models. The metrics for the 2020 data are summarized in Table 2 and show a similar trend as the 2022 data in Table 1.

**Fig. 7:**
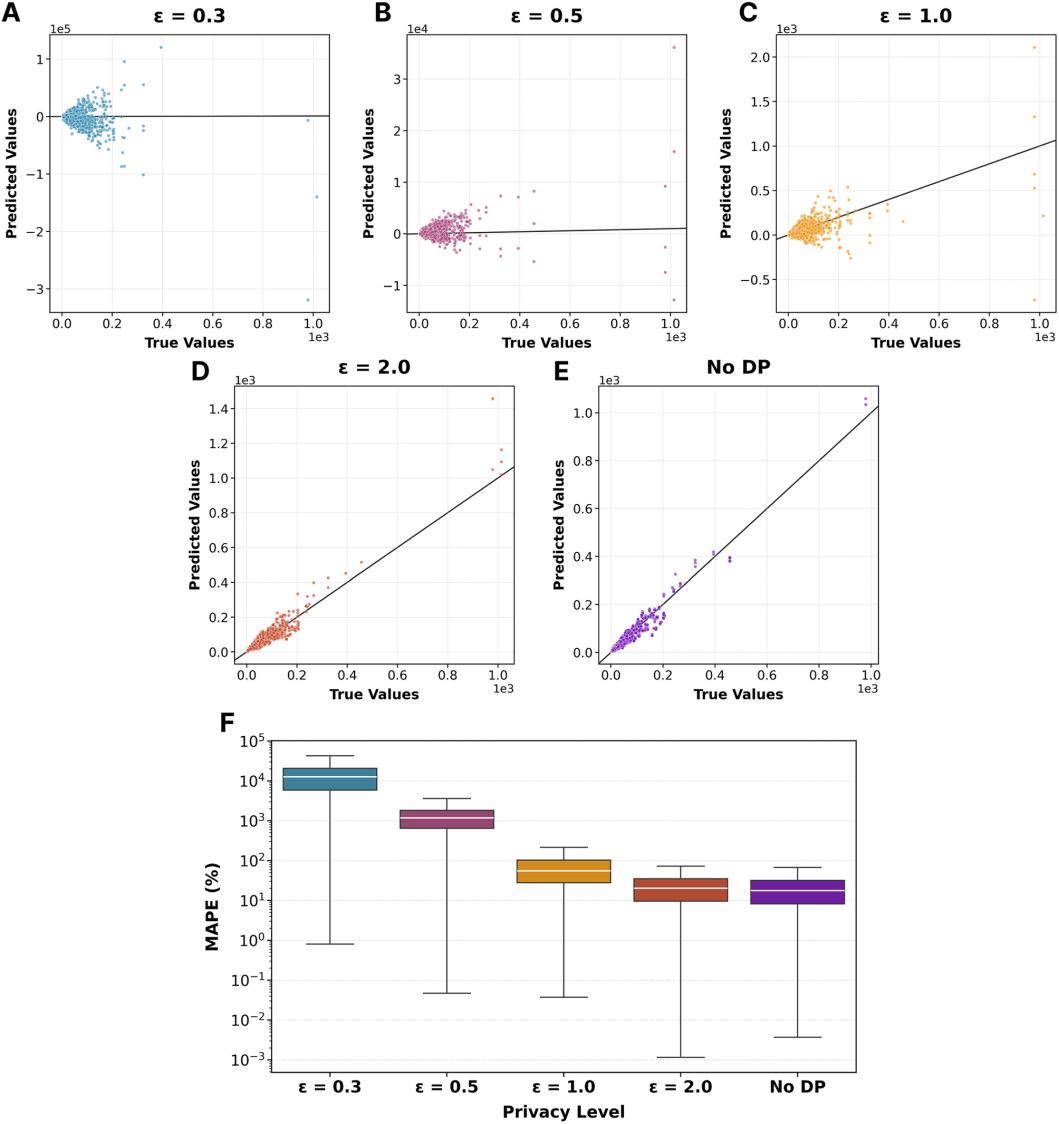
Comparison of prediction performance across different privacy levels for November 2020 data. Similar to Fig. 5, (**A**–**E**) show scatter plots of predicted versus true case counts for various privacy budgets, while Panel F displays box plots of MAPE for individual county predictions across all runs.

Similar to the 2022 results, we observe a clear degradation in model performance as the privacy budget decreases. At $\varepsilon = 0.3$, the model again produces essentially unusable predictions with extremely high MSE ($1.70 \times 10^8$) and deeply negative $R^2$ values (approximately $-40{,}100$). The MAPE values exceed 11,000%, indicating complete prediction failure. As shown in Fig. 7A, predictions at this privacy level appear randomly distributed with no correlation to actual case counts. At $\varepsilon = 0.5$, while still performing poorly, the model shows considerably better metrics than the equivalent privacy level in the 2022 data. The MSE drops to approximately $2.7 \times 10^6$, which represents a reduction of about two orders of magnitude compared to the 2022 results at the same privacy level ($1.12 \times 10^8$). The MAPE also shows relative improvement at about 1,500% versus 1,200% in the 2022 data. At $\varepsilon = 1.0$, the model achieves an $R^2$ of 0.07, indicating performance close to a mean-value predictor. This is substantially better than the corresponding 2022 model at the same privacy level ($R^2 = -1.72$). The MSE improves to 3,925, compared to around 721,000 in the 2022 data. Figure 7C shows predictions that begin to correlate with true values, but still with considerable dispersion. At $\varepsilon = 2.0$, the model achieves good performance with $R^2 = 0.93$, nearly matching the non-DP model. The MAE of 9.46 is only marginally higher than the non-DP value of 8.18, suggesting that at this privacy level, the added noise has limited impact on practical prediction quality. Figure 7D shows predictions that closely track the actual values across the entire range of case counts. The non-DP model establishes a slightly higher performance ceiling than in the 2022 data, with $R^2 = 0.96$ compared to 0.90. Also, the absolute errors are substantially lower in the 2020 data, with MAE of 8.18 against 87.69 in 2022, reflecting the lower absolute case numbers during this earlier pandemic phase. However, the MAPE is actually higher at 23.39% versus 18.15% in 2022, suggesting that despite lower absolute errors, the relative prediction accuracy may be somewhat worse.

The variance in model performance across multiple runs, as reflected in the standard deviations, follows similar patterns to the 2022 data, with extreme variability at low privacy budgets that stabilizes as $\varepsilon$ increases. Figure 7F illustrates this through the MAPE box plots, which show the distribution of individual percentage errors (MAPE) for each county across all runs. These box plots highlight the substantial outliers and wide distributions at $\varepsilon = 0.3$ and $\varepsilon = 0.5$ that contract substantially at higher privacy budgets.

In summary, the results for 2020 confirm the results from the 2022 data. Again, we were able to achieve good prediction performance at $\varepsilon = 2.0$, with the model closely matching the non-DP case but still preserving privacy guarantees.

**Table 2:** Performance metrics for COVID-19 case prediction on November 2020 data. Results from 15 runs, 75 federated rounds, and 30 local epochs per round.

$\varepsilon$	MSE	MAE	MAPE (%)	$R^2$
0.3	$1.70 \times 10^{8} \pm 3.15 \times 10^{8}$	$4.74 \times 10^{3} \pm 5.05 \times 10^{3}$	$1.11 \times 10^{4} \pm 1.12 \times 10^{4}$	$-4.01 \times 10^{4} \pm 7.43 \times 10^{4}$
0.5	$2.69 \times 10^{6} \pm 4.47 \times 10^{6}$	$647.46 \pm 557.15$	$1.51 \times 10^{3} \pm 1.19 \times 10^{3}$	$-6.33 \times 10^{2} \pm 1.06 \times 10^{3}$
1.0	$3.93 \times 10^{3} \pm 3.74 \times 10^{3}$	$29.56 \pm 13.26$	$69.85 \pm 28.72$	$0.07 \pm 0.88$
2.0	$296.03 \pm 117.12$	$9.46 \pm 1.25$	$25.61 \pm 3.22$	$0.93 \pm 0.03$
non-DP	$183.49 \pm 7.41$	$8.18 \pm 0.16$	$23.39 \pm 0.67$	$0.96 \pm 0.00$

### Privacy-utility tradeoff across epsilon values

To provide a more explicit view of the privacy-utility tradeoff, we conduct additional experiments on county-level data for both epidemic periods with a finer grid of privacy budgets,

$$\begin{aligned} \varepsilon \in \{0.2,0.3,0.4,0.5,0.75,1.0,1.5,2.0,3.0,5.0,\infty \} \end{aligned}$$

**Fig. 8:**
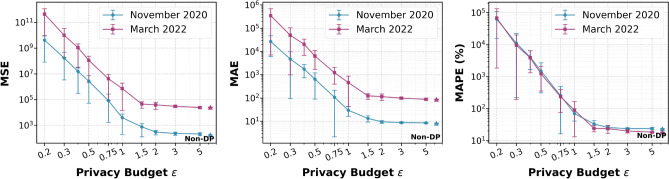
Privacy-utility tradeoff across privacy budgets. Privacy-utility curves for the November 2020 and March 2022 county-level datasets. Each panel shows mean ± standard deviation over 15 runs of (**A**) MSE, (**B**) MAE, and (**C**) MAPE as a function of the privacy budget $\varepsilon$. Both axes are on a logarithmic scale, and the non-DP baseline is indicated at the right-hand side of each curve.

We excluded $\varepsilon = 0.1$, because the RDP accountant produces prohibitively large noise multipliers at this extreme privacy level, which leads to completely unstable training. For each value of $\varepsilon$ we repeated the DP-FL training 15 times and recorded mean ± standard deviation of MSE, MAE, MAPE, and $R^2$. The resulting privacy-utility curves in Fig. 8 plot these metrics as a function of the privacy budget on logarithmic axes for November 2020 and March 2022. For very strict privacy ($\varepsilon \le 0.5$), the forecasts are essentially unusable, with MAPE values exceeding $1{,}000~\%$ and very large variability across runs. Between $\varepsilon = 0.5$ and $\varepsilon = 1.0$ the curves exhibit a sharp drop in all three error measures, reflecting the strongly nonlinear dependence of the RDP noise multiplier on $\varepsilon$. For moderately strong privacy ($\varepsilon \ge 2$), the DP-FL models in both periods closely approach the non-DP baseline and additional increases of $\varepsilon$ yield only marginal utility gains. The detailed summary statistics for all privacy budgets and both epidemic phases are reported in Table 4.

While we already provided $R^2$ values for each privacy budget, we additionally compared our FL models with a simple 7-day trailing moving average (MA7) baseline. This baseline is computed on the same test samples used in the main experiments – however without the FL setting and by the use of the full local information. For November 2020, the MA7 baseline achieves a MAPE of $24.2\%$, compared with $23.4\%$ for non-DP FL and $25.6\%$ for DP-FL at $\varepsilon =2.0$. For March 2022, the MA7 baseline achieves a MAPE of $20.0\%$, compared with $18.1\%$ for non-DP FL and $23.6\%$ for DP-FL at $\varepsilon =2.0$ (see Table 5 and 6). These results show that this naive baseline is already competitive in our short-horizon setting: the non-DP FL model performs similarly, while DP-FL at $\varepsilon = 2.0$ remains within a small margin of the naive baseline in both epidemic phases.

### Convergence across federated rounds

To analyze how predictive performance evolves during federated training under different privacy guarantees, we track the test error as a function of the number of federated rounds. For each epidemic period (November 2020 and March 2022) and for privacy budgets $\varepsilon \in \{0.5,1.0,2.0\}$, as well as the non-DP baseline, we train the model for 75 federated rounds with the same settings as in the main experiments (30 local epochs per round, clipping norm $S = 0.5$, and $\delta = 10^{-5}$). Every 5 rounds, we evaluate the current global model on the same test set and record the MAPE. We repeat this for 15 runs and report mean ± standard deviation across runs.

**Fig. 9:**
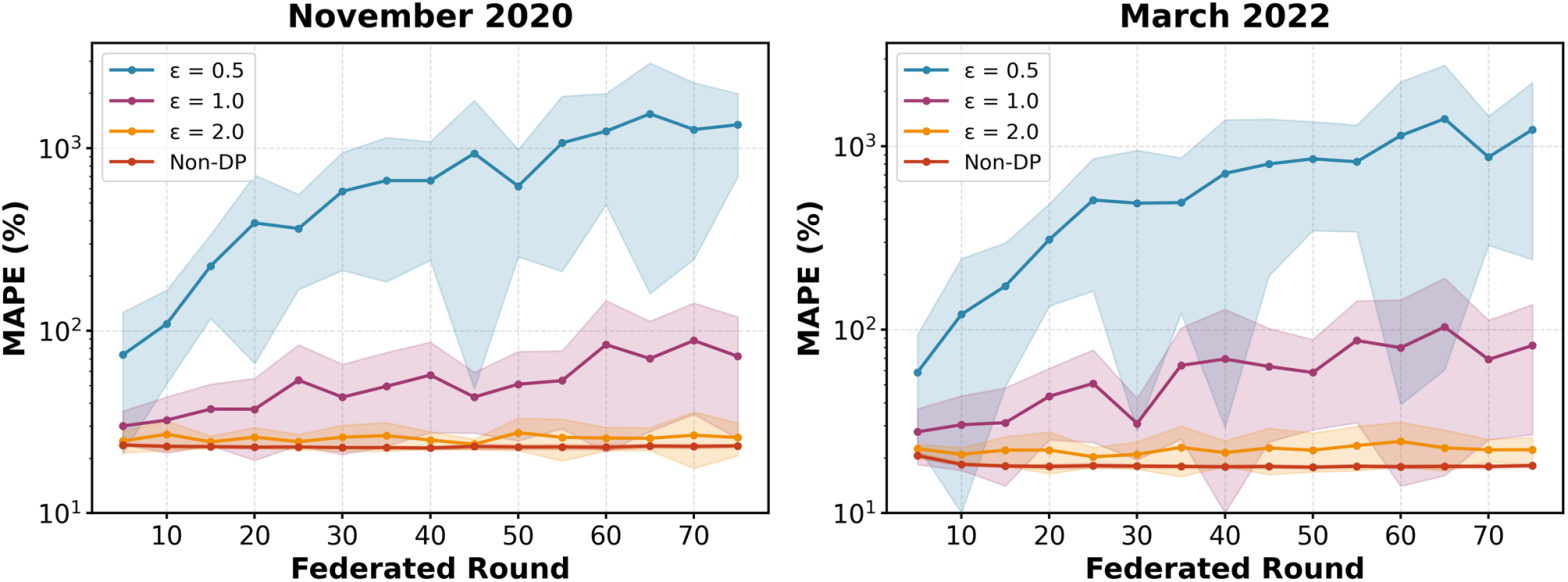
Convergence of DP-FL across federated rounds for different privacy budgets. Test MAPE (%) as a function of the number of federated rounds for November 2020 (left) and March 2022 (right). Curves show mean ± standard deviation over 15 runs for privacy budgets $\varepsilon = 0.5$ (blue), $\varepsilon = 1.0$ (purple), $\varepsilon = 2.0$ (orange), and Non-DP (red).

Figure 9 shows the resulting curves. For very strict privacy ($\varepsilon = 0.5$), the test MAPE is already very high after a few rounds (around 80-$100~\%$) and continues to increase over the course of training, with wide uncertainty bands that reflect large variability across runs. This indicates that the strong DP noise at this privacy level prevents stable learning and leads to increasingly noisy predictions. For intermediate privacy ($\varepsilon = 1.0$), the MAPE initially stabilizes (November 2020) or decreases slightly (March 2022) in the early rounds, but then stays at a much higher level than for $\varepsilon = 2.0$ (typically between 50 and $150~\%$) and shows noticeable variation across runs. This suggests that additional rounds provide only small improvements and can even worsen performance when training is continued for too long.

**Fig. 10:**
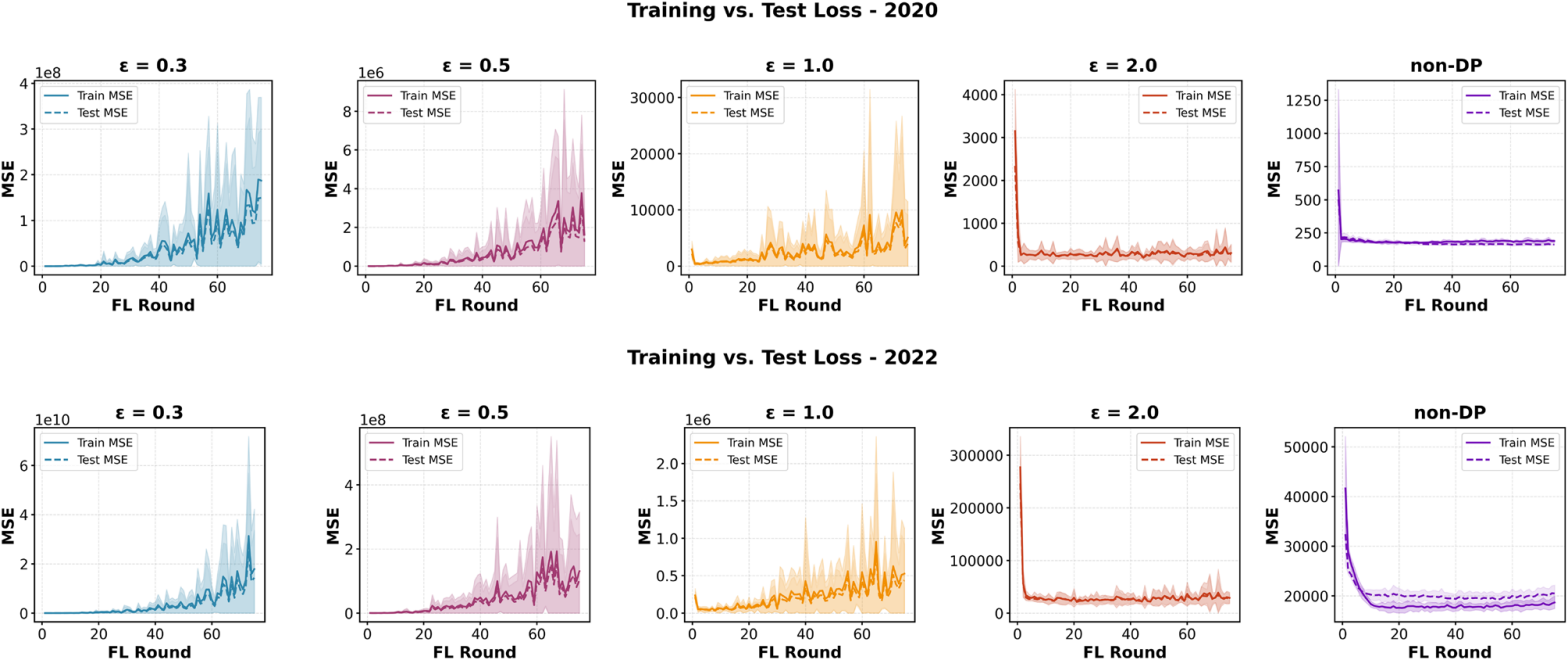
Training and test loss across federated rounds. Training vs. test MSE per FL round for all evaluated privacy budgets ($\varepsilon \in \{0.3, 0.5, 1.0, 2.0\}$ and non-DP). Solid lines show training MSE and dashed lines show test MSE, with shaded bands indicating ± standard deviation over 15 independent runs. The top row shows results for November 2020 and the bottom row for March 2022. The train/test gap remains small throughout all rounds with no systematic divergence. Absolute loss levels are higher for smaller $\varepsilon$ as expected, reflecting the regularization effect of DP noise.

In contrast, for $\varepsilon = 2.0$ the curves quickly stabilize on a plateau with low MAPE values (about $26~\%$ in November 2020 and about $22~\%$ in March 2022). The non-DP baseline converges slightly faster and to slightly lower final MAPE values (around $23~\%$ in November 2020 and about $18~\%$ in March 2022), but the gap to $\varepsilon = 2.0$ remains small in both epidemic phases. The small increase of the $\varepsilon = 2.0$ curve at the last evaluation point in March 2022 lies well within the uncertainty variability band. Overall, these convergence patterns support the conclusion that privacy budgets around $\varepsilon \approx 2$ allow the model to reach useful forecasts, whereas very strict privacy ($\varepsilon = 0.5$) leads to unstable training dynamics and unusable predictions. Training and test MSE converged in parallel across all privacy budgets and both epidemic phases, with no evidence of overfitting (see Fig. 10).

## Discussion

We developed a differential privacy-preserving federated learning approach for predicting COVID-19 case numbers on a spatially resolved level and validated the approach on county-level for two different time periods, November 2020 and March 2022. This was done to explore the balance between prediction accuracy and privacy-preservation in different stages of the pandemic. Our results demonstrate a clear relationship between privacy level (expressed through the privacy budget $\varepsilon$) and prediction quality.

Let us note that the novelty of this paper is not in presenting an MLP-based FL setup that universally outperforms simple or advanced forecasting rules and the comparison with the MA7 baseline shows that a simple baseline is competitive in our short-window setting. Our main contribution can be seen as the characterization of how much the predictive utility can be retained when client-level DP is imposed on a shared FL model; in particular, we identify a utility-based operating point under the evaluated DP accounting setup in the epidemic use case. The open-source publication of our full data and modeling pipeline can be seen as an additional contribution that allows other researchers to verify generalizations with their individual data and model structures. The strong performance of the MA7 baseline should be interpreted in light of the trailing 7-day moving average preprocessing. Since both the input values and the prediction target are trailing 7-day averages, the resulting time series are smoother and change more slowly than raw daily counts. However, we also evaluated larger and more advanced neural architectures in exploratory checks, including wider MLPs and LSTM variants without DP (see Table 5) and larger MLP variants with DP at $\varepsilon =2.0$ (see Table 6). We note that these architecture checks are single-run diagnostics to contextualize the basic model choice. These results support our decision to retain the simple MLP architecture in the main DP-FL experiments.

The baseline comparison should be interpreted in the context of the study focus. The MA7 baseline was included to provide a simple, causal, and directly interpretable reference for the short-window forecasting task. In particular, we did not compare against conventional time-series forecasting methods such as ARIMA or Prophet, against a centralized model trained on pooled county-level data, or against DP-SGD-based training. These baselines could change the absolute level of predictive performance and may also affect how attractive a given privacy budget appears in practice. A systematic comparison with such forecasting and privacy-preserving learning baselines should therefore be addressed in future work.

One goal of our study was to explore the possibility of localized predictions at a finer spatial resolution than the county-level. Although we developed a probabilistic approach to disaggregate county data to the community-level, the resulting data proved inadequately representative of actual local dynamics. The random assignment of cases to communities based solely on population data likely disrupted local spread patterns, resulting in time series dominated by unstructured noise rather than learnable transmission dynamics. In contrast, real-world community data, while noisy, retains the causal structure of infection chains which FL can leverage. This experience underscores the challenges of working with sensitive health data at a highly resolved spatial level. While protecting privacy is a key concern, the availability of granular data is crucial for precise localized predictions. Due to the lack of case data resolved on community-level with often only several hundreds of individuals, we could not test our approach on very small population units. Future research will thus consider the creation of a more realistic synthetic dataset. This dataset should follow the structural patterns as observed during the COVID-19 pandemic on community-level. Furthermore, it should be created such that it can be published for scientific research. To do so, we will reach out to public health experts with access to more finely resolved data series.

Our findings on county-level confirm the fundamental tradeoff between privacy and utility that is characteristic of differential privacy implementations. At very strict privacy guarantees ($\varepsilon = 0.3$ and $\varepsilon = 0.5$), predictions are practically unusable, as evidenced by extremely high MSE values and negative $R^2$ values. This observation aligns with the theoretical foundation of differential privacy, where a lower $\varepsilon$ value leads to stronger noise addition to model parameters.

For a privacy-preserving budget of $\varepsilon = 2.0$, we were able to achieve predictions that closely match the quality of non-DP models, with an $R^2$ value of 0.85 for the 2022 data and 0.93 for the 2020 data. The MAPE was around 24% for the 2022 data and 26% for the 2020 data, indicating that while there is some degradation in prediction quality compared to non-DP models, the results are still practically useful. This suggests that, under the evaluated DP accounting setup, useful predictive performance can be retained at a moderate privacy budget.

The identified operating point near $\varepsilon \approx 2$ was consistent across both epidemic phases studied, despite substantially different signal magnitudes (November 2020 vs. March 2022). This consistency should, however, be interpreted within the experimental scope of this work. All primary county-level experiments are based on one country (Germany), one disease (COVID-19), two one-month epidemic periods, one seven-day forecasting horizon, one primary MLP architecture, and one client-level DP-FL configuration. The precise location of the operating point depends on the FL configuration and application setting. While the formal privacy guarantee is determined by the accountant parameters, the empirically useful privacy budget may shift for different model classes, datasets, diseases, forecast horizons, client definitions, clipping norms, sampling rates, or training schedules. The value $\varepsilon \approx 2$ should therefore not be interpreted as a generally optimal privacy budget for epidemiological forecasting. Researchers applying this framework in a different setting should re-evaluate the tradeoff for their specific configuration. An important contribution of this paper is the open-source publication of the entire data processing and modeling workflow, which allows other researchers and public health experts to insert individual data sets and model structures. In particular, we provided an extensive read-me with detailed steps for reproduction and generalization.

Adopting $\varepsilon = 2.0$ as a practical operating point has concrete privacy implications that deserve explicit discussion. At the client level, the DP guarantee bounds an adversary’s ability to determine whether a given LHA participated in the federation. Concretely, for any event *O*, the probability of observing *O* under two neighboring federated runs, one with and one without a given LHA, can differ by at most a multiplicative factor $e^\varepsilon$ plus $\delta$. Increasing $\varepsilon$ from 0.3 to 2.0 therefore substantially weakens this indistinguishability guarantee, and we do not interpret $\varepsilon =2$ as a negligible privacy loss. The guarantee should be read as a formal bound under the stated client-level DP model, not as an empirical measurement of attack success. It directly concerns the influence of an LHA’s participation on the released aggregated model; it does not by itself quantify the success probability of concrete attacks such as membership inference. Risks outside the trusted-server threat model, including access to individual pre-aggregated updates, are discussed separately below. In weighing this privacy-utility tradeoff, it is important to note that pandemic mitigation relies on timely and accurate data. We regard $\varepsilon =2$ as a practically relevant candidate in this setting because stricter privacy budgets yielded unusable forecasts, whereas $\varepsilon =2$ retained useful predictive performance. Public health experts and decision makers have to make legal and ethical considerations to find an appropriate operational tradeoff to minimize privacy loss while protecting the entire population as well as the individual against the threat of infection. At the same time, the protected unit in our setting is an entire LHA dataset rather than an individual record, and we do not claim that $\varepsilon =2$ alone establishes regulatory acceptability. Practical compliance depends on the full technical and governance context. Translating $(\varepsilon ,\delta )$-DP into a concrete success probability for a specific membership inference attack requires additional assumptions about the adversary, prior knowledge, and attack protocol^42^, so we refrain from reporting a single universal attack-success number here. Empirical evaluation of membership inference attacks against the federated model under our specific setting is an important direction for future work to further characterize the practical privacy risk.

German counties vary substantially in population size (from under 50,000 to over 3.5 million inhabitants), which raises concerns about whether smaller counties might be disadvantaged in a federated learning setting. To address this, we conducted an analysis stratifying model performance by population clusters. The results show that while smaller counties exhibit higher absolute errors due to lower case counts, the privacy-utility tradeoff remains relatively uniform across all population sizes. Importantly, smaller counties benefit from the global model by learning temporal patterns that would be undetectable in their local data alone. The gradient clipping mechanism in our DP-FL algorithm ($S=0.5$) prevents larger counties from dominating the aggregation, ensuring that updates from all counties contribute equally.

We note that using MSE on raw, unnormalized case counts inherently emphasizes counties and epidemic periods with larger absolute case numbers. This choice is relevant for predicting absolute public-health burden, but it means that larger counties contribute more strongly to the squared-error objective. The L2 clipping at $S=0.5$ mitigates this at the update level but does not eliminate the effect on the learned representation. We tested MSE on log-transformed counts in preliminary experiments and observed no relevant improvement in predictive performance, consistent with the gradient clipping already acting as an implicit scale normalization. The relative performance metrics (MAPE and MdAPE) across population clusters further support that no strong systematic skew toward larger counties is present in our results. Nevertheless, the privacy-utility tradeoff reported here is tied to the chosen raw-count regression objective and evaluation metrics. Alternative formulations, including population-normalized targets, weighted losses, and count-based likelihoods, could lead to different utility estimates and possibly different privacy-budget conclusions. A systematic evaluation of such population-normalized and count-likelihood formulations is therefore an important direction for future work.

We base our experiments on an established client-level DP-FL setup to isolate and interpret the impact of the privacy budget on forecast quality. Prior work has proposed a range of refinements, including local-DP federated learning with per-client privacy modules^28^, algorithms that study different locations for adding noise and analyze their convergence and utility guarantees^26^, and healthcare-focused FL frameworks^31–34^. These methods target algorithmic improvements and can in principle be combined with our setting. We expect that these algorithms may further improve forecast accuracy; a systematic evaluation of such variants for infectious disease dynamics should be included in future work. Regarding the model choice, we utilized a standard MLP to demonstrate the impact of the privacy mechanism; however, the framework is compatible with any gradient-based model. We additionally evaluated more advanced neural architectures without DP (see Table 5), including wider MLPs and LSTM variants, under the same county-level forecasting setup. We note that these architecture checks are single-run diagnostics to contextualize the basic model choice. These results support our decision to retain the simple MLP architecture in the main DP-FL experiments. We consider this work as a proof of concept, with more work required before this methodology can be used as intended.

Our setup follows a central, trusted-curator DP model for federated learning, in which the coordinator receives clipped client updates, aggregates them, and applies the Gaussian mechanism to the aggregated update. We do not implement secure aggregation in the present experiments. Therefore, the coordinator can observe individual pre-aggregated clipped updates, and the framework does not provide protection against an honest-but-curious server. If such a server attempted gradient inversion or reconstruction from observed client updates, these attacks would fall outside our current privacy guarantee, which is defined for the released aggregated model under the trusted-server assumption. In a realistic multi-LHA deployment, this assumption would require the coordinating server to be operated by a trusted public institution, such as a national or regional public health body, under a shared institutional and regulatory framework. If this trust assumption is not acceptable, the mechanism should be combined with additional safeguards such as secure aggregation^43^.

A limitation of our study concerns the quality and resolution of the underlying data. Clearly, it would have been better if we could also have validated our approach on the much smaller communities. However, this data was unavailable and for a generated synthetic data set, we could not validate whether the noisy results are representative or not. Although we attempted to mitigate weekend effects on county-level by applying a 7-day moving average, these artifacts partially remain and limit the model’s learning ability. The raw, unsmoothed case counts are dominated by a systematic weekly reporting artifact, as reported counts drop by roughly a factor of two to three on weekends and spike correspondingly at the start of the week when backlogged reports are registered. Critically, this artifact is heterogeneous across counties: some counties report consistently on weekends while others do not, so the pattern differs between clients in a way that is unrelated to disease dynamics and cannot be generalized by a federated model. The smoothed signal is therefore the appropriate prediction target, as it represents the underlying epidemic trend rather than administrative reporting conventions. Eventually, the problem of underreporting prevails: the reported case numbers represent only a fraction of actual infections with differences across spatial levels. According to data from the Robert Koch Institute^39^, test positivity rates in 2022 ranged between 30 % and 50 %, suggesting a substantial dark figure. However, we assume that the underreporting rate remains relatively constant within the short timeframes considered. Future research should incorporate auxiliary data sources, such as wastewater-based indicators or dark figure estimators, to better account for true infection numbers.

The tradeoff between the need for detailed data for precise predictions and privacy requirements underscores the relevance of our FL approach with DP, which could support LHAs in using their granular data for collaborative learning without directly sharing it.

As an important outcome for public health, we conclude that with an appropriate privacy budget ($\varepsilon = 2.0$), DP-FL models can generate predictions that nearly match the quality of non-DP models. This supports the feasibility of privacy-preserving collaboration between LHAs under the stated assumptions without heavily affecting the quality of decision support. It should not be interpreted as a deployment-ready privacy solution: questions of attack resistance beyond the stated threat model, secure aggregation, governance, regulatory interpretation, and operational implementation remain for future work.

Overall, our work demonstrates that privacy-preserving federated learning approaches are suitable in principle for predicting infectious disease dynamics if the privacy budget is carefully chosen.

## Methods

We develop a FL scheme with client-level DP to produce short-term local case forecasts while keeping raw data decentralized. Our goal is to provide a formal client-level DP guarantee for the released aggregated model under the stated threat model. For instance, in scenarios involving collaborative LHAs, our goal is to bound the influence of each LHA’s participation on the released model. An adversary who observes only the released aggregated model should not be capable of discerning whether any client’s data has been included in the federated run (within $\varepsilon$ and $\delta$ bounds). DP provides plausible deniability not only for groups of a client’s samples but also for any client participating in the federated run. Consequently, any adverse privacy impact on clients (or their training samples) cannot be directly linked to their participation in the protocol run under this observation model.

### Data preprocessing

Our objective is time-series forecasting of local case counts using data which is reported at the local level. We construct supervised learning examples by using a historical window of size $H=10$ days over each administrative unit’s time series and predicting the case count $P=7$ days ahead. The window size *H* and prediction horizon *P* were determined empirically to ensure robust predictive performance. Let $c_{k,t}$ denote the raw, unnormalized number of daily reported cases for geographic unit *k*, $k=1,2,3,\ldots$ on day *t*. Therefore, the input vector

1 $$\begin{aligned} \textbf{x}_{k,t} = \bigl [c_{k,t-H+1},\dots ,\,c_{k,t}\bigr ] \end{aligned}$$

contains the reported daily cases on the preceding *H* days, and the target

2 $$\begin{aligned} y_{k,t} = c_{k,t+P} \end{aligned}$$

is the case count *P* days into the future. Missing reports are handled as zero reported cases. To mitigate weekly reporting artifacts (e.g., inconsistent weekend reporting), we applied a trailing 7-day moving average, where the smoothed value at day *t* is the mean of days $t-6$ through *t*. This causal formulation uses only past and current observations and avoids any look-ahead bias. This smoothing is crucial not only for noise reduction but also to stabilize gradient updates in the FL process. For model evaluation, we construct examples by using a historical window of $H = 10$ consecutive days and predicting the case count $P = 7$ days ahead for each county. Within each epidemic period (November 2020 and March 2022) and for each county separately, we order all resulting input-label pairs by the date of the prediction target and assign the earliest $90~\%$ to the training set and the most recent $10~\%$ to the test set. In this way, all test samples correspond to later days than the training samples for the same county, reflecting the intended short-term forecasting use case. The November 2020 and March 2022 datasets are handled in separate experiments, i.e. models are trained and evaluated independently on each period. After splitting, we remove the location identifier from the feature vectors since they should not influence the model in any way.

### Model architecture

We use PyTorch^44^ to build a fully connected MLP as our prediction model. The architecture is consistent across both spatial resolutions, community- and county-based; see the *Results* section for details on the datasets used. The input to the network is a vector of length *H* containing the *H* most recent daily case counts; with zero reported cases counting as daily case count. This vector is propagated through three fully connected hidden layers of sizes 128, 64, and 32 neurons, each followed by a rectified linear unit (ReLU) activation function. A final linear output unit produces the predicted case count *P* days ahead. We use the mean squared error (MSE) as the loss function, which is minimized by the Adam optimizer with a learning rate of 0.001.

The MLP was intentionally chosen as a simple, well-understood architecture rather than selected by an extensive model selection process as, e.g., in Schmidt et al.^10^. The primary goal of this work is to characterize the effect of the privacy budget $\varepsilon$ on forecast quality in the federated setting. A simple model of fixed size serves this purpose well: it isolates the effect of the DP mechanism without introducing additional sources of variation such as model complexity or architecture-specific optimization behavior. To the best of our knowledge, DP had not yet been studied thoroughly with federated learning for the important application of epidemic forecasting. Nevertheless, the DP-FL framework is architecture-agnostic and applies to any gradient-based model. As the full data transformation and modeling pipeline has been published open-source, researchers are capable of testing generalizations with their individual data sets and model architectures.

### Differential privacy with federated learning

We use a federated learning approach with client-level differential privacy. To formally define our privacy guarantees, we rely on the standard definition of $(\varepsilon , \delta )$-differential privacy^24^. In the following, we briefly summarize the background of differential privacy with our model choices.

### Differential privacy

#### Definition 1

A randomized mechanism ${\mathcal {M}}$ with range ${\mathcal {R}}$ satisfies $(\varepsilon , \delta )$-differential privacy, if for any two adjacent datasets *D* and $D'$, i.e., $D'=D \cup \{x\}$ for some *x* in the data domain (or vice versa), and for any subset of outputs $O \subseteq {\mathcal {R}}$, it holds that

3 $$\begin{aligned} \Pr [{\mathcal {M}}(D) \in O] \le e^\varepsilon \Pr [{\mathcal {M}}(D') \in O] + \delta \end{aligned}$$

Intuitively, DP guarantees that an adversary, provided with the output of $\mathcal {M}$, can draw almost the same conclusions (up to $\varepsilon$ with probability larger than $1 - \delta$) about any group no matter if it is included in the input of $\mathcal {M}$ or not^24^. This means, for any group owner, a privacy breach is unlikely to be due to its participation in the dataset.

We use the Gaussian mechanism to upper bound privacy leakage when releasing aggregated model updates from the server.

#### Definition 2

(Gaussian Mechanism^24^) Let $f: {\mathbb {R}}^n \rightarrow \mathbb {R}^d$ be an arbitrary function that maps *n*-dimensional input to *d* logits with sensitivity being:

4 $$\begin{aligned} S= \max _{D,D'} \Vert f(D)-f(D')\Vert _2 \end{aligned}$$

over all adjacent datasets *D* and $D' \in {\mathcal {D}}$. The Gaussian Mechanism $\mathcal {M}_\sigma$, parameterized by $\sigma$, adds noise to the output, i.e.,

5 $$\begin{aligned} \mathcal {M_\sigma }(x) = f(x) + \mathcal {N}(0,\sigma ^2 I). \end{aligned}$$

**Adversarial Model.** We assume a trusted central server that faithfully performs aggregation and noise addition and does not attempt to inspect or reconstruct individual client updates. The passive honest-but-curious adversary considered here controls a subset of clients, not the server. Secure aggregation is not implemented in the current protocol. Consequently, individual pre-aggregated clipped updates are visible to the trusted server, and the framework does not provide protection against an honest-but-curious server. Attacks that require server-side inspection of such updates, including gradient inversion or reconstruction attacks, are therefore outside the current threat model. The client-level DP guarantee stems from Gaussian noise added to the aggregated update, while clipping is used to bound sensitivity. We further assume a controlled internal communication setting in which only participating clients and the trusted server have access to the communication channels. Protection against external eavesdroppers, for example via transport encryption, is orthogonal to our contribution.

**Privacy Model.** In federated learning, the notion of *adjacent (neighboring) datasets* used in DP generally refers to pairs of datasets differing by one client (*client-level* DP), or by one group of one user (*group-level* DP), or by one data point of one user (*record-level* DP). Our approach focuses on the *client-level* DP, where each client corresponds to a data holder, such as an LHA. This granularity is the natural choice for our setting: each LHA holds the complete time series for its county, and the privacy guarantee should protect the LHA’s participation as a whole, not individual records. Server-side noise addition following^25,27^ is used rather than local DP, as it requires less noise for equivalent guarantees under the trusted-server model. The adversary considered in this privacy guarantee observes the released aggregated model or global model updates, not individual pre-aggregated client updates. Under this observation model, the adversary should not be capable of discerning whether any client’s data has been included in the federated run (within $\varepsilon$ and $\delta$ bounds). We believe this adversarial model applies in many practical scenarios where private information extends across multiple samples within a client’s training data (for example, the presence of a group of samples, such as individuals with a specific feature). Differential Privacy provides plausible deniability not only for groups of a client’s samples but also for any client participating in the federated run. Consequently, any adverse privacy impact on clients (or their training samples) cannot be directly linked to their participation in the protocol run under this observation model. The detailed privacy analysis, including the accounting via Rényi Differential Privacy (RDP), is provided in the supplementary material.

#### DP-FL algorithm

The core methodology employs FL with client-level DP, summarized in Algorithm 1. Our client-level DP-FL protocol is conceptually aligned with established DP-FL schemes^25,26^, where each selected client clips its local update to a fixed sensitivity, and the server adds Gaussian noise to the averaged update with a noise multiplier chosen via the Rényi DP accountant. Following standard practices in differentially private federated learning^31^, the clipping threshold *S* and the client sampling rate *q* were determined empirically. For reproducibility and to allow verification of the $(\varepsilon ,\delta )$-DP guarantees, we list all relevant hyperparameter values here. The county-level experiments use $N=400$ clients (one per German county), of which on average $m=100$ are selected per round, giving a sampling rate of $q = m/N = 0.25$. Training runs for $T=75$ federated rounds with $E=30$ local epochs per round. The clipping norm is $S=0.5$ and the privacy failure probability is $\delta =10^{-5}$. The noise multiplier *c* is computed for each target $\varepsilon$ via the RDP accountant; the resulting values are included in Table 3. All hyperparameters are additionally explained in Table 3. The training process basically follows four steps:

1. **Client selection:** In each round *t*, a subset $\mathbb {K}$ of clients is selected from the total number of clients $N_{\textrm{total}}$. Specifically, each client is independently included with probability
  $$q \;=\; \frac{m}{N_{\textrm{total}}},$$
  so that on average *m* participants (i.e., geographic units) contribute updates per round. This random sampling strategy reduces both communication and computational load. It also represents slow or unavailable clients, since each round depends only on the selected clients. When DP is enabled, this subsampling also amplifies privacy by lowering the effective per-round privacy loss.
2. **Local training:** Then, each selected client $k \in \mathbb {K}$ receives the current global model weights $\textbf{w}_{i-1}$. The client then performs local training for a fixed number of epochs *E* on its own local dataset which remains local at all times. After local training completes, resulting in local model weights $\textbf{w}_{i-1}^k$, the client *k* computes the local model update $\Delta \textbf{w}_i^k = \textbf{w}_{i-1}^k - \textbf{w}_{i-1}$ relative to the initial given global model. Only this model update is sent to the server, ensuring that no privacy-critical data leaves the local client.
3. **DP mechanism:** When client-level DP is enabled (i.e., $\varepsilon <\infty$), each participating client first applies the Euclidean 2-norm bound on its model update $\Delta \textbf{w}_i^k$ by clipping to a fixed sensitivity $S=0.5$:
  $$\Delta \widehat{\textbf{w}}_i^k \;=\;\frac{\Delta \textbf{w}_i^k}{\max \!\bigl (1,\Vert \Delta \textbf{w}_i^k\Vert _2/S\bigr )}.$$
  The server then aggregates these clipped updates by summation and divides by the expected batch size *m* to obtain the mean update. Finally, Gaussian noise $\mathcal {N}(0,\sigma ^2 \textbf{I})$ is added to this averaged update, where
  $$\sigma \;=\;\frac{S\cdot c}{m},$$
  and the noise multiplier *c* is chosen via the Rényi DP (RDP) accountant^45^. If privacy is disabled ($\varepsilon =\infty$), no clipping or noise is applied and the raw updates $\Delta \textbf{w}_i^k$ are simply averaged.
4. **Global model update:** Let
  $$\Delta \overline{\textbf{w}}_i \;:=\; \frac{1}{m}\sum _{k\in \mathbb {K}} \Delta \widehat{\textbf{w}}_i^{\,k}$$
  denote the mean clipped update. In the DP case, we draw $\xi _i \sim \mathcal {N}(0,\sigma ^2 \textbf{I})$. Then, the server updates the global model by adding the final averaged update:
  $$\textbf{w}_i = \textbf{w}_{i-1} + \Delta \overline{\textbf{w}}_i + \xi _i$$
  In the non-DP case, this reduces to: $\textbf{w}_i = \textbf{w}_{i-1} + \Delta \overline{\textbf{w}}_i$.

After $T_{\textsf{cl}}$ rounds, the final global model is evaluated on the test dataset. To account for variability due to random client selection and noise addition, the entire training procedure is repeated multiple times with different random seeds.

**Algorithm 1:**
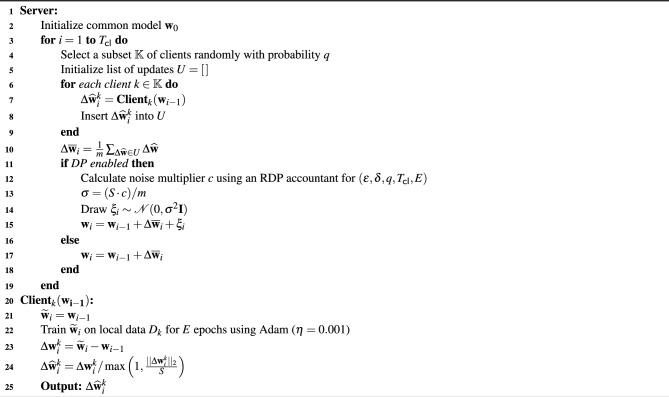
Federated learning with client-level privacy.

**Table 3:** Overview of model hyperparameters.

Symbol		Description		Value	
*H*		Input window length (days)		10	
*P*		Prediction horizon (days)		7	
*E*		Local training epochs per round		30	
*N*		Total number of clients		400	
*m*		Expected clients selected per round		100	
$T_{\textsf{cl}}$		Total number of FL rounds		75	
*q*		Client sampling rate (*m*/*N*)		0.25	
*S*		L2 clipping norm		0.5	
$\delta$		Privacy failure probability		$10^{-5}$	
$\eta$		Learning rate (Adam)		0.001	
$\varepsilon$		DP budget		{0.3, 0.5, 1.0, 2.0,$\infty$}	
*c*		Noise multiplier (via RDP accountant)		see below	
Noisemultipliers *c* computed by the RDP accountant for each evaluated privacy budget ($N=400$,$m=100$,$q=0.25$,$T=75$,$\delta =10^{-5}$):					

$\varepsilon$	0.3	0.5	1.0	2.0	$\infty\:\:$
*c*	27.50	16.88	8.98	4.88	0

**Table 4:** Performance metrics across privacy budgets. Results from 15 runs and 75 federated rounds for different $\varepsilon$ values. All values are mean ± standard deviation.

Year	$\varepsilon$	MSE	MAE	MAPE (%)	$R^2$
Nov 2020	0.20	$4.18\textrm{e}{+09} \pm 5.31\textrm{e}{+09}$	$2.67\textrm{e}{+04} \pm 2.07\textrm{e}{+04}$	$6.25\textrm{e}{+04} \pm 4.67\textrm{e}{+04}$	$-9.87\textrm{e}{+05} \pm 1.25\textrm{e}{+06}$
Nov 2020	0.30	$1.70\textrm{e}{+08} \pm 3.15\textrm{e}{+08}$	$4.74\textrm{e}{+03} \pm 5.05\textrm{e}{+03}$	$1.11\textrm{e}{+04} \pm 1.12\textrm{e}{+04}$	$-4.01\textrm{e}{+04} \pm 7.43\textrm{e}{+04}$
Nov 2020	0.40	$1.52\textrm{e}{+07} \pm 1.83\textrm{e}{+07}$	$1.76\textrm{e}{+03} \pm 1.02\textrm{e}{+03}$	$4.08\textrm{e}{+03} \pm 2.40\textrm{e}{+03}$	$-3.59\textrm{e}{+03} \pm 4.31\textrm{e}{+03}$
Nov 2020	0.50	$2.69\textrm{e}{+06} \pm 4.47\textrm{e}{+06}$	$6.47\textrm{e}{+02} \pm 5.57\textrm{e}{+02}$	$1.51\textrm{e}{+03} \pm 1.19\textrm{e}{+03}$	$-6.33\textrm{e}{+02} \pm 1.06\textrm{e}{+03}$
Nov 2020	0.75	$8.37\textrm{e}{+04} \pm 1.84\textrm{e}{+05}$	$1.08\textrm{e}{+02} \pm 1.09\textrm{e}{+02}$	$2.55\textrm{e}{+02} \pm 2.39\textrm{e}{+02}$	$-1.88\textrm{e}{+01} \pm 4.34\textrm{e}{+01}$
Nov 2020	1.00	$3.93\textrm{e}{+03} \pm 3.74\textrm{e}{+03}$	$2.96\textrm{e}{+01} \pm 1.33\textrm{e}{+01}$	$6.98\textrm{e}{+01} \pm 2.87\textrm{e}{+01}$	$7.33\textrm{e}{-02} \pm 8.82\textrm{e}{-01}$
Nov 2020	1.50	$7.60\textrm{e}{+02} \pm 6.31\textrm{e}{+02}$	$1.32\textrm{e}{+01} \pm 3.96\textrm{e}{+00}$	$3.32\textrm{e}{+01} \pm 9.01\textrm{e}{+00}$	$8.21\textrm{e}{-01} \pm 1.49\textrm{e}{-01}$
Nov 2020	2.00	$2.96\textrm{e}{+02} \pm 1.17\textrm{e}{+02}$	$9.46\textrm{e}{+00} \pm 1.25\textrm{e}{+00}$	$2.56\textrm{e}{+01} \pm 3.22\textrm{e}{+00}$	$9.30\textrm{e}{-01} \pm 2.76\textrm{e}{-02}$
Nov 2020	3.00	$2.33\textrm{e}{+02} \pm 6.43\textrm{e}{+01}$	$8.75\textrm{e}{+00} \pm 7.13\textrm{e}{-01}$	$2.36\textrm{e}{+01} \pm 1.65\textrm{e}{+00}$	$9.45\textrm{e}{-01} \pm 1.52\textrm{e}{-02}$
Nov 2020	5.00	$2.14\textrm{e}{+02} \pm 5.47\textrm{e}{+01}$	$8.54\textrm{e}{+00} \pm 6.58\textrm{e}{-01}$	$2.40\textrm{e}{+01} \pm 1.84\textrm{e}{+00}$	$9.50\textrm{e}{-01} \pm 1.29\textrm{e}{-02}$
Nov 2020	Non-DP	$1.83\textrm{e}{+02} \pm 7.41\textrm{e}{+00}$	$8.18\textrm{e}{+00} \pm 1.63\textrm{e}{-01}$	$2.34\textrm{e}{+01} \pm 6.67\textrm{e}{-01}$	$9.57\textrm{e}{-01} \pm 1.75\textrm{e}{-03}$
Mar 2022	0.20	$4.41\textrm{e}{+11} \pm 7.26\textrm{e}{+11}$	$3.50\textrm{e}{+05} \pm 3.43\textrm{e}{+05}$	$6.78\textrm{e}{+04} \pm 6.59\textrm{e}{+04}$	$-1.66\textrm{e}{+06} \pm 2.73\textrm{e}{+06}$
Mar 2022	0.30	$9.97\textrm{e}{+09} \pm 2.41\textrm{e}{+10}$	$4.97\textrm{e}{+04} \pm 5.47\textrm{e}{+04}$	$9.62\textrm{e}{+03} \pm 1.04\textrm{e}{+04}$	$-3.75\textrm{e}{+04} \pm 9.07\textrm{e}{+04}$
Mar 2022	0.40	$1.08\textrm{e}{+09} \pm 1.06\textrm{e}{+09}$	$2.04\textrm{e}{+04} \pm 1.36\textrm{e}{+04}$	$3.90\textrm{e}{+03} \pm 2.57\textrm{e}{+03}$	$-4.08\textrm{e}{+03} \pm 3.97\textrm{e}{+03}$
Mar 2022	0.50	$1.12\textrm{e}{+08} \pm 1.24\textrm{e}{+08}$	$6.37\textrm{e}{+03} \pm 4.66\textrm{e}{+03}$	$1.23\textrm{e}{+03} \pm 8.75\textrm{e}{+02}$	$-4.20\textrm{e}{+02} \pm 4.67\textrm{e}{+02}$
Mar 2022	0.75	$4.17\textrm{e}{+06} \pm 4.98\textrm{e}{+06}$	$1.23\textrm{e}{+03} \pm 8.33\textrm{e}{+02}$	$2.37\textrm{e}{+02} \pm 1.61\textrm{e}{+02}$	$-1.47\textrm{e}{+01} \pm 1.88\textrm{e}{+01}$
Mar 2022	1.00	$7.21\textrm{e}{+05} \pm 1.19\textrm{e}{+06}$	$4.60\textrm{e}{+02} \pm 4.17\textrm{e}{+02}$	$8.99\textrm{e}{+01} \pm 7.68\textrm{e}{+01}$	$-1.72\textrm{e}{+00} \pm 4.50\textrm{e}{+00}$
Mar 2022	1.50	$4.49\textrm{e}{+04} \pm 2.37\textrm{e}{+04}$	$1.24\textrm{e}{+02} \pm 3.54\textrm{e}{+01}$	$2.42\textrm{e}{+01} \pm 5.45\textrm{e}{+00}$	$8.31\textrm{e}{-01} \pm 8.92\textrm{e}{-02}$
Mar 2022	2.00	$3.88\textrm{e}{+04} \pm 2.19\textrm{e}{+04}$	$1.15\textrm{e}{+02} \pm 3.53\textrm{e}{+01}$	$2.36\textrm{e}{+01} \pm 6.69\textrm{e}{+00}$	$8.54\textrm{e}{-01} \pm 8.26\textrm{e}{-02}$
Mar 2022	3.00	$2.99\textrm{e}{+04} \pm 5.50\textrm{e}{+03}$	$9.82\textrm{e}{+01} \pm 9.91\textrm{e}{+00}$	$1.99\textrm{e}{+01} \pm 2.43\textrm{e}{+00}$	$8.87\textrm{e}{-01} \pm 2.07\textrm{e}{-02}$
Mar 2022	5.00	$2.40\textrm{e}{+04} \pm 3.72\textrm{e}{+03}$	$8.77\textrm{e}{+01} \pm 5.79\textrm{e}{+00}$	$1.83\textrm{e}{+01} \pm 6.60\textrm{e}{-01}$	$9.09\textrm{e}{-01} \pm 1.40\textrm{e}{-02}$
Mar 2022	Non-DP	$2.52\textrm{e}{+04} \pm 1.30\textrm{e}{+03}$	$8.77\textrm{e}{+01} \pm 2.22\textrm{e}{+00}$	$1.81\textrm{e}{+01} \pm 3.58\textrm{e}{-01}$	$9.05\textrm{e}{-01} \pm 4.88\textrm{e}{-03}$

**Table 5:** Non-DP architecture comparison with unscaled and standardized training. All models predict 7-day-ahead county-level case counts directly. Metrics are evaluated on the original count scale in a single run.

Period	Model	Scale	MSE	MAE	MAPE (%)	$R^2$
Nov. 2020	MLP [128, 64, 32]	unscaled	257.27	8.29	23.50	0.947
Nov. 2020	MLP [256, 128, 64]	unscaled	265.13	8.40	23.97	0.945
Nov. 2020	MLP [512, 256, 128, 64]	unscaled	267.02	8.37	23.87	0.945
Nov. 2020	LSTM 32	unscaled	4679.47	21.68	35.24	0.032
Nov. 2020	LSTM 64	unscaled	4193.45	18.33	36.43	0.132
Nov. 2020	LSTM 128	unscaled	3908.83	16.42	35.88	0.191
Nov. 2020	LSTM 64 (2 layers)	unscaled	4151.18	18.96	40.77	0.141
Nov. 2020	MLP [128, 64, 32]	standardized	902.81	12.32	49.51	0.813
Nov. 2020	MLP [256, 128, 64]	standardized	999.15	12.72	50.60	0.793
Nov. 2020	MLP [512, 256, 128, 64]	standardized	1210.58	13.55	52.61	0.749
Nov. 2020	LSTM 32	standardized	2992.76	13.83	48.89	0.381
Nov. 2020	LSTM 64	standardized	2614.67	12.88	41.75	0.459
Nov. 2020	LSTM 128	standardized	2378.78	12.66	40.05	0.508
Nov. 2020	LSTM 64 (2 layers)	standardized	3361.50	15.44	49.03	0.304
Mar. 2022	MLP [128, 64, 32]	unscaled	17356.36	78.12	16.19	0.930
Mar. 2022	MLP [256, 128, 64]	unscaled	17937.94	79.08	16.30	0.928
Mar. 2022	MLP [512, 256, 128, 64]	unscaled	19367.87	81.46	16.62	0.922
Mar. 2022	LSTM 32	unscaled	493375.19	494.51	84.61	-0.981
Mar. 2022	LSTM 64	unscaled	428212.62	425.23	67.65	-0.720
Mar. 2022	LSTM 128	unscaled	344442.31	329.50	50.61	-0.383
Mar. 2022	LSTM 64 (2 layers)	unscaled	412461.44	407.55	63.89	-0.656
Mar. 2022	MLP [128, 64, 32]	standardized	156157.56	173.54	38.33	0.373
Mar. 2022	MLP [256, 128, 64]	standardized	176060.41	190.49	44.20	0.293
Mar. 2022	MLP [512, 256, 128, 64]	standardized	173275.69	188.18	42.11	0.304
Mar. 2022	LSTM 32	standardized	113954.96	152.90	37.62	0.542
Mar. 2022	LSTM 64	standardized	109846.21	150.50	34.10	0.559
Mar. 2022	LSTM 128	standardized	108935.71	156.34	34.29	0.563
Mar. 2022	LSTM 64 (2 layers)	standardized	113789.13	150.43	33.86	0.543
7-day moving average baseline: MAPE = 24.23% (Nov. 2020), 19.98% (Mar. 2022).

**Table 6:** Selected MLP architectures evaluated without and with DP at $\varepsilon =2.0$ under the same county-level forecasting setup. Metrics are evaluated on the original count scale in a single run.

Period	Model	$\varepsilon$	MSE	MAE	MAPE (%)	$R^2$
Nov. 2020	MLP [128, 64, 32]	Non-DP	262.39	8.16	23.61	0.946
Nov. 2020	MLP [128, 64, 32]	2.0	379.30	11.22	25.48	0.922
Nov. 2020	MLP [256, 128, 64]	Non-DP	212.52	7.79	23.26	0.956
Nov. 2020	MLP [256, 128, 64]	2.0	249.88	8.82	23.33	0.948
Nov. 2020	MLP [512, 256, 128, 64]	Non-DP	198.04	8.09	25.16	0.959
Nov. 2020	MLP [512, 256, 128, 64]	2.0	1167.07	17.54	38.63	0.758
Mar. 2022	MLP [128, 64, 32]	Non-DP	18786.14	82.67	17.32	0.925
Mar. 2022	MLP [128, 64, 32]	2.0	23324.65	93.70	18.49	0.906
Mar. 2022	MLP [256, 128, 64]	Non-DP	18912.42	79.85	16.30	0.924
Mar. 2022	MLP [256, 128, 64]	2.0	32408.91	109.86	23.59	0.870
Mar. 2022	MLP [512, 256, 128, 64]	Non-DP	20954.20	86.61	17.09	0.916
Mar. 2022	MLP [512, 256, 128, 64]	2.0	35982.92	111.93	21.74	0.855

## Supplementary Information

Supplementary Information.

## Data Availability

All data used in this study are publicly available from the Robert Koch Institute (RKI) and can be accessed via the MEmilio-epidata Python package^40^. Step-by-step instructions for downloading the data and reproducing all results are provided in ^46^.
