# Supplementary material for: Differentially private federated learning for localized control of infectious disease dynamics
Source: Sci Rep. 2026 Jul 6;16:20808. doi: 10.1038/s41598-026-60725-1 (PMC13338152; doi:10.1038/s41598-026-60725-1)
Supplement: Supplementary file 1 — Supplementary Information. [file 41598_2026_60725_MOESM1_ESM.pdf]

Supplementary Information

Privacy Analysis

The privacy analysis of our differentially private baseline is discussed here. As in[1,2], we consider the Sampled Gaussian Mechanism (SGM) —a composition of subsampling and the additive Gaussian noise (defined in Definition 3)— for privacy amplification. Moreover, we first compute the SGM's Renyi Differential Privacy as in[2] and then we use the conversion Theorem 3 from[3] to switch back to Differential Privacy.

Definition 1 (Rényi divergence) *Let P and Q be two distributions on $\mathscr{X}$ defined over the same probability space, and let p and q be their respective densities. The Rényi divergence of a finite order $\alpha \neq 1$ between P and Q is defined as follows:*

$$D_\alpha (P \parallel Q) \triangleq \frac{1}{\alpha - 1} \ln \int_{\mathscr{X}} q(x) \left(\frac{p(x)}{q(x)} \right)^\alpha dx \, .$$

Rényi divergence at orders $\alpha = 1, \infty$ is defined by continuity.

Definition 2 (Rényi differential privacy (RDP)) *A randomized mechanism $\mathscr{M} : \mathscr{D} \to \mathscr{R}$ satisfies (α, ρ)-Rényi differential privacy (RDP) if for any two adjacent inputs D, $D' \in \mathscr{D}$ it holds that*

$$D_\alpha \left(\mathscr{M}(D) \parallel \mathscr{M}(D') \right) \leq \rho$$

In this work, we call two datasets D, D' to be adjacent if $D' = D \cup \{x\}$ (or vice versa).

Definition 3 (Sampled Gaussian Mechanism (SGM)) *Let f be an arbitrary function mapping subsets of $\mathscr{D}$ to $\mathbb{R}^d$. We define the Sampled Gaussian mechanism (SGM) parametrized with the sampling rate $0 < q \leq 1$ and the noise $\sigma > 0$ as*

$$\mathrm{SG}_{q,\sigma} \triangleq f\left(\{x : x \in D \text{ is sampled with probability } q\}\right) + \mathscr{N}(0, \sigma^2 \mathbb{I}^d),$$

where each element of D is independently and randomly sampled with probability q without replacement.

As for the Gaussian Mechanism, the Sampled Gaussian Mechanism consists of adding identically and independently distributed Gaussian noise with zero mean and variance σ^2 to each coordinate value of the true output of f. In fact, the Sampled Gaussian Mechanism draws vector values from a multivariate spherical (or isotropic) Gaussian distribution which is described by random variable $\mathscr{N}(0, \sigma^2 \mathbb{I}^d)$, where d is omitted if it is unambiguous in the given context.

Analysis

The privacy guarantee of our approach is quantified using the revisited moment accountant[2] that restates the moments accountant introduced in[1] using the notion of Rényi differential privacy (RDP) defined in[4].

Let μ_0 denote the probability density function (pdf) of $\mathscr{N}(0, \sigma^2)$ and let μ_1 denote the pdf of $\mathscr{N}(1, \sigma^2)$. Let μ be the mixture of two Gaussians $\mu = (1-q)\mu_0 + a\mu_1$, where q is the sampling probability of a single record in a single round.

Theorem 1 [2]. *Let $\mathrm{SG}_{q,\sigma}$ be the Sampled Gaussian mechanism for some function f and under the assumption $\Delta_2 f \leq 1$ for any adjacent $D, D' \in \mathscr{E}$. Then $\mathrm{SG}_{q,\sigma}$ satisfies (α, ρ)-RDP if*

$$\rho \leq \frac{1}{\alpha - 1} \log \max(A_\alpha, B_\alpha) \tag{1}$$

where $A_\alpha \triangleq \mathbb{E}_{z \sim \mu_0}[(\mu(z)/\mu_0(z))^\alpha]$ and $B_\alpha \triangleq \mathbb{E}_{z \sim \mu}[(\mu_0(z)/\mu(z))^\alpha]$

Theorem 1 states that applying SGM to a function of sensitivity at most 1 (which also holds for larger values without loss of generality) satisfies (α,ρ)-RDP if $\rho \leq \frac{1}{\alpha-1}\log(\max\{A_\alpha, B_\alpha\})$. Thus, analyzing RDP properties of SGM is equivalent to upper bounding A_α and B_α.

From Corollary 7. in[2], $A_\alpha \geq B_\alpha$ for any $\alpha \geq 1$. Therefore, we can reformulate Eq. (1) as

$$\rho \leq \xi_{\mathcal{N}}(\alpha|q) := \frac{1}{\alpha-1}\log A_\alpha \tag{2}$$

To compute A_α, we use the numerically stable computation approach proposed in[2] (Sec. 3.3) depending on whether α is expressed as an integer or a real value.

Theorem 2 (Composability[4]) *Suppose that a mechanism $\mathscr{M}$ consists of a sequence of adaptive mechanisms $\mathscr{M}_1, \ldots, \mathscr{M}_k$ where $\mathscr{M}_i : \prod_{j=1}^{i-1}\mathscr{R}_j \times \mathscr{E} \to \mathscr{R}_i$. If all the mechanisms in the sequence are (α,ρ)-RDP, then the composition of the sequence is $(\alpha, k\rho)$-RDP.*

In particular, Theorem 2 holds when the mechanisms themselves are chosen based on the (public) output of the previous mechanisms. By Theorem 2, it suffices to compute $\xi_{\mathcal{N}}(\alpha|q)$ at each step and sum them up to bound the overall RDP privacy budget of an iterative mechanism composed of DP mechanisms obtained over different steps.

Theorem 3 (Conversion from RDP to DP[3]) *If a mechanism $\mathscr{M}$ is (α,ρ)-RDP then it is $((\rho + \log((\alpha-1)/\alpha) - (\log\delta + \log\alpha)/(\alpha-1), \delta)$-DP for any $0 < \delta < 1$.*

Theorem 4 (Privacy of our approach) *For any $0 < \delta < 1$ and $\alpha \geq 1$, our approach is $(\min_\alpha(T_{\text{cl}} \cdot \xi(\alpha|q) + \log((\alpha-1)/\alpha) - (\log\delta + \log\alpha)/(\alpha-1)), \delta)$-DP, where $\xi_{\mathcal{N}}(\alpha|q)$ is defined in Eq. (2).*

The proof follows from Theorems 1 to 3 and the fact that a client (LHA) is sampled in every federated round with a probability of q.

References

1. Abadi, M. *et al.* Deep learning with differential privacy. In *Proceedings of the 2016 ACM SIGSAC conference on computer and communications security*, 308–318 (2016).
2. Mironov, I., Talwar, K. & Zhang, L. R\'enyi differential privacy of the sampled gaussian mechanism (2019).
3. Balle, B., Barthe, G., Gaboardi, M., Hsu, J. & Sato, T. Hypothesis testing interpretations and renyi differential privacy. In *International Conference on Artificial Intelligence and Statistics*, 2496–2506 (PMLR, 2020).
4. Mironov, I. Rényi differential privacy. In *2017 IEEE 30th computer security foundations symposium (CSF)*, 263–275 (IEEE, 2017).
